# Bioactive Functional Nanolayers of Chitosan–Lysine Surfactant with Single- and Mixed-Protein-Repellent and Antibiofilm Properties for Medical Implants

**DOI:** 10.1021/acsami.1c01993

**Published:** 2021-05-17

**Authors:** Urban Ajdnik, Lidija Fras Zemljič, Olivija Plohl, Lourdes Pérez, Janja Trček, Matej Bračič, Tamilselvan Mohan

**Affiliations:** † Faculty of Mechanical Engineering, Institute of Engineering Materials and Design, Laboratory for Characterization and Processing of Polymers, University of Maribor, Smetanova ulica 17, 2000 Maribor, Slovenia; ‡ Department of Surfactants and Nanobiotechnology, Institute for Advanced Chemistry of Catalonia (IQAC-CSIC), Jordi Girona 18-26, 08034 Barcelona, Spain; § Faculty of Natural Sciences and Mathematics, Department of Biology, University of Maribor, Koroška cesta 160, 2000 Maribor, Slovenia; ∥ Institute for Chemistry and Technology of Biobased Systems (IBioSys), Graz University of Technology, Stremayrgasse 9, 8010 Graz, Austria

**Keywords:** silicone implants, protein-repellent, antimicrobial, chitosan, lysine, bioactive coatings, adsorption, QCM-D

## Abstract

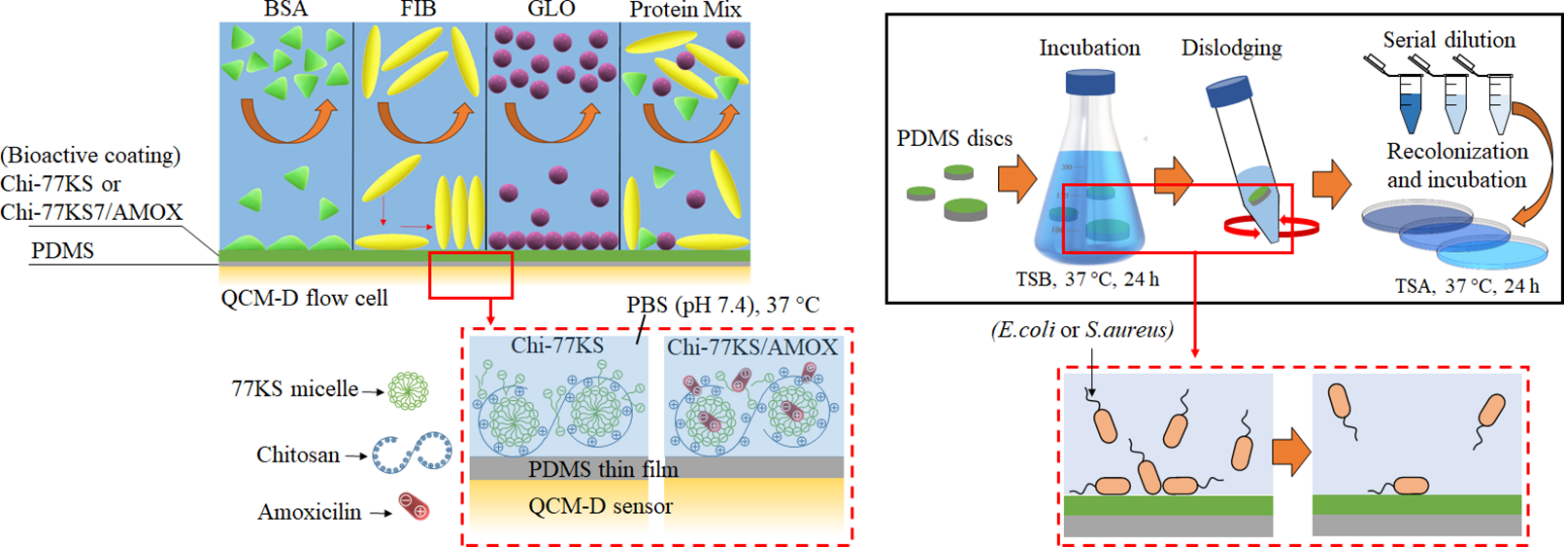

Medical implant-associated infections resulting from biofilm formation triggered by unspecific protein adsorption are the prevailing cause of implant failure. However, implant surfaces rendered with multifunctional bioactive nanocoatings offer a promising alternative to prevent the initial attachment of bacteria and effectively interrupt biofilm formation. The need to research and develop novel and stable bioactive nanocoatings for medical implants and a comprehensive understanding of their properties in contact with the complex biological environment are crucial. In this study, we developed an aqueous stable and crosslinker-free polyelectrolyte–surfactant complex (PESC) composed of a renewable cationic polysaccharide, chitosan, a lysine-based anionic surfactant (77KS), and an amphoteric antibiotic, amoxicillin, which is widely used to treat a number of infections caused by bacteria. We successfully introduced the PESC as bioactive functional nanolayers on the “model” and “real” polydimethylsiloxane (PDMS) surfaces under dynamic and ambient conditions. Besides their high stability and improved wettability, these uniformly deposited nanolayers (thickness: 44–61 nm) with mixed charges exhibited strong repulsion toward three model blood proteins (serum albumin, fibrinogen, and γ-globulin) and their competitive interactions in the mixture in real-time, as demonstrated using a quartz crystal microbalance with dissipation (QCM-D). The functional nanolayers with a maximum negative zeta potential (ζ: −19 to −30 mV at pH 7.4), water content (1628–1810 ng cm^–2^), and hydration (low viscosity and elastic shear modulus) correlated with the mass, conformation, and interaction nature of proteins. In vitro antimicrobial activity testing under dynamic conditions showed that the charged nanolayers actively inhibited the growth of both Gram-negative (*Escherichia coli*) and Gram-positive (*Staphylococcus aureus*) bacteria compared to unmodified PDMS. Given the ease of fabrication of multifunctional and charged biobased coatings with simultaneous protein-repellent and antimicrobial activities, the limitations of individual approaches could be overcome leading to a better and advanced design of various medical devices (e.g., catheters, prosthetics, and stents).

## 1 Introduction

The use of implantable medical devices is a common and indispensable part of medical care for diagnostic and therapeutic purposes. Invasive medical devices inserted through the body orifices pose a considerable risk as they provide bacteria access from external environment.^1^ Medical devices are incomparably more prone to contamination than human tissue, as the colonization of medical devices requires an approximately 10,000 times lower amount of bacteria.^2^ Biofilms bring about up to 80% of microbial infections^2^ and are challenging to eliminate due to their pervasiveness and resilience.^3^ Medical device-related microbial infections (MDMI) worsen patient’s quality of life and increase mortality, while at the same time press a heavy financial burden on the healthcare system.^2^ Furthermore, an ageing population, rising disease prevalence, and deteriorating lifestyle drive the expansionary use of medical devices, projected to reach $133 billion in 2022.^2,4^ On top of that, antimicrobial resistance poses a challenging global problem.^4^ Preventing the initial steps toward MDMI is vital to avert the spread of infections worldwide. Following the insertion, blood proteins and interstitial fluids immediately cover the implant surface,^1,2^ starting with the most abundant high-mobility protein serum albumin, followed by fibrinogen and globulins.^5^ Protein adsorption is a complex process, governed by the protein properties, environmental conditions (pH and temperature), and surface properties.^6^ Proteins adsorb to the surface through hydrophobic, van der Waals, electrostatic interactions, and hydrogen bonding in different conformations, orientations, densities, and quantities^6^ to expose more potential binding sites and increase the surface activity.^6^ Proteins adsorbed on the biomaterial surface postimplantation offer numerous potential binding sites for bacterial adhesion. Therefore, the study and fundamental understanding of protein adsorption at the beginning of the biofilm formation due to their role in subsequent attachment of cells and bacteria are essential.^7^

According to the current research, the best possible treatment for biofilm-based infections is to prevent the initial attachment of microorganisms, inhibit their growth, and disrupt biofilm formation already at the beginning.^2,8^ An effective implant must be biocompatible with a functional surface that is antifouling as well as antimicrobial. Cationic substances [e.g*.*, quaternary ammonium compounds, biopolymer chitosan (Chi), and antimicrobial peptides], enzymes, and silver ions have already demonstrated the effective antimicrobial activity on surfaces.^9^ However, these substances bear limitations. While the application of individual agents commonly exhibits poor antibiofilm performance and still allows unspecific protein adsorption, their mode of action stems from the interactions with pathogen’s cell membrane (e.g., physical degradation or disruption of specific components of the cell membrane).^10^ To summarize, there are no medical devices that meet all of the above-mentioned criteria. This fact further emphasizes the need to research and develop novel bioactive coatings for medical implants and a comprehensive understanding of their properties. It is critical to ensure that medical devices in contact with the complex biological environment have functional interfaces, successfully repel proteins, and further prevent bacterial adhesion. Accordingly, multifunctional biobased coatings using a combination of methods to prevent biofilm formation could provide a superior solution to improve and overcome the limitations of individual approaches.^11^

Recently, it has been of high interest to combine naturally occurring polysaccharides (PSs) with other substances to obtain synergistic formulations with improved functionality and higher efficiency. PSs comprise an important component of life matter and combine excellent biocompatibility and biodegradability, which are typical features of polymers used in biomaterials. On top of all, PSs are naturally abundant, nontoxic, and affordable materials, used in an expansive variety of applications.^12^ The combination of PSs as polyelectrolytes (inexpensive and biocompatible) and surfactants in the form of a bioactive nanocoating on medical devices is still less explored and interesting. Such combinations lead to the formation of various structures, such as micelles, complexes, precipitates, and gels that can serve as a drug delivery system.^13^ Polyelectrolyte–surfactant complexes (PESCs) play a vital role in the pharmaceutical, cosmetic, and food industries.^14,15^ It was already reported that the use of natural or modified PSs such as Chi, hyaluronic acid, and alginate enables flexible design of bioactive coatings with improved antibacterial and protein-repelling properties to protect surfaces from infections.^16,17^ However, the studies combining the PSs and surfactants as a bioactive coating toward biofilm formation are scarce.

In this study, the emphasis is given on a comprehensive evaluation of the surface parameters, adsorption kinetics, and conformational changes of proteins and antibiofilm properties of innovative bioactive coatings, prepared by combining cationic Chi and oppositely charged surfactant 77KS (Chi-77KS) in the form of PESC as well as a nanocarrier for model drug amoxicillin (AMOX; Chi-77KS/AMOX). A comprehensive understanding of protein adsorption is an essential and crucial step for their role in subsequent attachment of cells, bacteria, and biofilm formation,^7^ and can be monitored by a quartz crystal microbalance with dissipation (QCM-D). It provides information on mass and structural changes associated with the processes on the sensor surface, as well as data about surface behavior, thickness, deposition rate, mechanical responses, and growth of layers in real-time, accompanied by viscoelastic phenomena and mathematical modeling.^18^ Several studies focused on protein-repelling surfaces in the initial steps using a single protein.^19−23^ We focused on the time-dependent frequency (Δ*f*) and dissipation (Δ*D*) change to reveal adsorption and desorption kinetics of bovine serum albumin (BSA), fibrinogen (FIB), γ-globulin (GLO), common proteins that participate in the formation of a biofilm layer after implant insertion, and their mixture under the flow conditions. Dissipation versus frequency (Δ*D*/Δ*f*) analyses were employed to reveal the viscoelastic properties, connected to the protein adhesion and detachment. All measurements were performed in phosphate-buffered saline (PBS) at pH 7.4 and 37 °C to mimic the physiological conditions of the human body. This is the first time that the adsorption of individual proteins BSA, FIB, GLO, and their mixture was studied using QCM-D on an innovative bioactive coating consisting of Chi and 77KS.

In our previous study,^24^ we prepared, adsorbed, and characterized Chi-77KS and Chi-77KS/AMOX on a model thin polydimethylsiloxane (PDMS) film. However, so far, there were no studies reported on the in situ implementation of bioactive coatings Chi-77KS and Chi-77KS/AMOX on a representative implant material and on multiprotein adsorption using QCM-D. A systematic study of protein adsorption on thin PDMS films was followed by an antibiofilm assay on casted PDMS discs using Gram-negative *Escherichia coli* (*E. coli*) and Gram-positive *Staphylococcus aureus* (*S. aureus*). Accordingly, we transferred the obtained knowledge from the QCM-D adsorption study on “model” samples directly to the “real” casted PDMS samples to improve and progress the development of bioactive coatings on medical devices and assist to pave a new way to study bioactive surfaces.

## 2 Experimental Section

### 2.1 Materials

An anionic surfactant derived from lysine (77KS)^24^ was supplied by the Institute for Advanced Chemistry of Catalonia IQAC-CSIC (Barcelona, Spain). Low-molecular-weight chitosan (Chi: 50,000–190,000 Da, 75–85% deacetylated), amoxicillin (AMOX: potency ≥900 μg mg^–1^), glacial acetic acid (AcOH: ≥99.7%), toluene, PBS, HCl (37%, ACS reagent), KCl (puriss. p.a., ≥99.5%), NaCl (≥99.0%, ACS reagent) and NaOH (5 M, pro analysis), bovine serum albumin (BSA: lyophilized powder, ≥96%), fibrinogen (FIB: 90% clottable), γ-globulin (GLO: ≥97%), deuterium oxide (D_2_O, 99.9 atom % D), poly(ethylene glycol) methyl ether thiol (PEG-SH:Mn2000), PDMS monomer, and a curing agent (SYLGARD 184) were supplied by Sigma-Aldrich, Austria. Ultrapure water (resistivity of 18.2 MΩ cm at 25 °C) was prepared using the Milli-Q system (Millipore Corporation, Massachusetts, USA). Gold-coated quartz crystal sensors QSX301 were purchased from Biolin Scientific (Gothenburg, Sweden). Gram-positive *S. aureus* ATCC 29213 and Gram-negative *E. coli* ATCC 25922 were provided by the American Type Culture Collection and maintained in the Laboratory of Microbiology at the Department of Biology, the University of Maribor, Slovenia. Micro agar (prod no. M1002.1000) was purchased from Duchefa Biochemie (Haarlem, The Netherlands), Difco tryptic soy agar (TSA) from Becton, Dickinson and Company (Le Pont de Claix, France), tryptic soy broth (TSB) from Biolife Italiana Srl (Milan, Italy), and ethanol (96% V/V, puriss.) from Honeywell (Seelze, Germany).

### 2.2 Sample Preparation

#### 2.2.1 Preparation of the Chi-77KS PESC (with and without Drug Incorporation)

The preparation of water-based PESC from Chi and 77KS incorporated with the AMOX drug was described in detail in our previous publication.^24^ Briefly, the Chi solution was prepared by dissolving 0.4 g of Chi in ultrapure water (∼180 mL) and with the addition of 0.4 mL AcOH. The mixture was stirred overnight with a magnetic stirrer at room temperature to ensure complete dissolution. Afterward, the pH was adjusted to 4.5 using 5.0 M NaOH and diluted to 200 mL, giving a final 2.0 g L^–1^ Chi solution. The 77KS was dissolved in ultrapure water at a concentration of 0.04 M (the pH of that solution was 8.3). The Chi-77KS PESC complex with a pH value of 6.5 was prepared by mixing Chi with 77KS to give a final Chi:77KS mass ratio of 1.0:2.4. The Chi-77KS/AMOX complex (pH of 6.5) was prepared using the same procedure as described above for the Chi-77KS complex with the prior addition of a 2.7 g L^–1^ concentration of AMOX to the prepared 0.04 M 77KS solution. The mass ratio of Chi:77KS/AMOX for the prepared PESC complex was 2.7:6.3:1.0. The solution of AMOX and 77KS is still homogenous at the AMOX concentration used.

#### 2.2.2 Preparation and Functionalization of PDMS Substrates with Chi-77KS and Chi 77KS/AMOX Nanolayers

##### 2.2.2.1 Preparation and Functionalization of “Model” PDMS Surfaces

The QCM-D Au-sensors were used for PDMS model film preparation. Briefly, the PDMS monomer and the curing agent in a 10 (monomer):1 (curing agent) ratio were dissolved in toluene to obtain a 10% (w/w) stock solution. The final 0.5% (w/w) PDMS solution was prepared by diluting the stock solution with toluene. 30 μL of the final PDMS solution was spin coated using a spin coater (POLOS MCD200, APT, Bienenbüttel, Germany) onto Au-sensors at 4000 revolutions per minute (rpm), an acceleration of 2500 rpm s^–1^, and 30 s at 25 °C.^25^ The films were then treated at 70 °C for 2 h. The adsorption of the Chi-77KS and Chi-77KS/AMOX complex PDMS “model” surface was performed using QCM-D at 25 ± 0.1 °C and a flow rate *Q* = 0.1 mL min^–1^. The detailed description can be found in our recent publication.^24^

##### 2.2.2.2 Preparation and Functionalization of “Real” PDMS Surfaces

“Real” samples in the form of PDMS discs (*A* = 1.2 cm^2^, *h* = 1.5 mm) were prepared by mixing the PDMS monomer and the curing agent in a 10 (monomer):1 (curing agent) ratio, followed by a subsequent treatment at 70 °C for 2 h. Chi-77KS and the Chi-77KS/AMOX complex were coated on a PDMS disc using the dip-coating method^26^ and subsequently dried with N_2_.

#### 2.2.3 Preparation of Protein Samples for QCM-D Experiments

BSA (1 mg mL^–1^), FIB (1 mL^–1^), and GLO (1 mg mL^–1^) were dissolved in PBS buffer prepared by dissolving one tablet per 200 mL of water (yielding 10 mM phosphate buffer, 2.7 mM potassium chloride, and 137 mM sodium chloride at pH 7.4) at room temperature. A mixed protein solution was prepared by mixing BSA, FIB, and GLO at the same concentrations as above in the PBS buffer at pH 7.4. All protein solutions and buffers were freshly prepared before the QCM-D measurements.

### 2.3 Atomic Force Microscopy

Surface topography and roughness parameters of the neat and functionalized PDMS “model” surfaces with Chi-77KS and Chi-77KS/AMOX coatings were characterized using a Keysight 7500 AFM multimode scanning probe microscope (Keysight Technologies, Santa Barbara, CA). The images were scanned in tapping mode with silicon cantilevers (ATEC-NC, Nanosensors, Germany) at an ambient temperature in air (a resonance frequency of 210–490 kHz and a force constant of 12–110 N m^–1^). All images were recorded with a resolution of 2048 × 2048 pixels and were processed using the freeware Gwyddion allowing for the AFM roughness to be calculated as the root mean square (R_rms_) deviation from the mean height of the topography after leveling of the images by mean plane subtraction.^27^

### 2.4 Water Contact Angle Measurements

The surface wettability of neat PDMS before and after functionalization with Chi-77KS and Chi-77KS/AMOX was investigated through Static Water Contact Angle SCA(H_2_O) measurements using an OCA 35 Optical Contact Angle Meter and SCA 20 (version 4.1.12) software (DataPhysics Instruments, Filderstadt, Germany). Each SCA(H_2_O) value was determined within 2 s of contact with the surface and is the average of at least six liquid droplets per surface. Two independent surfaces were used for each sample. Measurements were performed in triplicate at 25 °C, using a 3 μL drop of ultrapure water (ultrapure water, a resistivity of 18.2 MΩ cm at 25 °C).

### 2.5 Attenuated Total Reflection-Fourier Transform Infrared Spectroscopy and X-ray Photoelectron Spectroscopy

The attenuated total reflection-Fourier transform infrared (ATR-FTIR) spectra were measured using a PerkinElmer Spectrum GX Series-73565 spectrometer equipped with a diamond crystal ATR module. The scans were recorded in the range of 4000–400 cm^–1^ by 32 scans with a resolution of 4 cm^–1^. The elemental composition of the samples and the neat PDMS before and after coating with Chi-77KS and Chi-77KS/AMOX was determined using a TFA X-ray photoelectron spectroscopy (XPS) instrument from Physical Electronics GmbH (Feldkirchen/Münich, Germany). The ultimate pressure in the XPS chamber was 6 × 10^–8^ Pa. The samples were exposed to X-rays from monochromatic Al Kα_1,2_ radiation at 1486.6 eV. The diameter of an analysis area was 400 μm. Survey-scan spectra were measured at a pass energy of 187.85 eV with a 0.40 eV energy step. The elemental composition was determined using MultiPak v8.1c software from Physical Electronics, which was supplied with the spectrometer.

### 2.6 Streaming Potential Measurements

The surface ζ-potential was determined from the measurement of streaming current using the instrument SurPASS 3 (Anton Paar GmbH, Austria) and an Adjustable Gap Cell for 14 mm discs. The streaming current measurement was used as an alternative to streaming potential measurement because of the additional conductivity caused by the Au QCM-D sensor.^28^ A pair of QCM-D sensors with the same upper coating was fixed on the sample holders (with a circular cross section and a diameter of 14 mm), using double-sided adhesive tape with weak adhesion to ease the removal of the QCM-D sensors after completing the measurement series. The distance between adjacent sensor discs was adjusted to 103 ± 4 μm during several rinsing step cycles with the 10 mM KCl aqueous solution. The streaming current was measured with an Ag/AgCl electrode. The ζ-potential as a function of pH was determined in an aqueous solution of 10 mM NaCl (the ionic strength was high enough to suppress any contribution of interfacial conductivity according to Jachimska et al*.*^28^ During the pH scan measurement, the pH was adjusted with 0.05 M HCl and 0.05 M NaOH. By means of surface ζ-potential analyses, the aqueous electrolyte solution was purged with N_2_.

### 2.7 Quartz Crystal Microbalance with Dissipation

A QCM-D instrument (model E4) from Q-Sense (Gothenburg, Sweden) was used. The instrument simultaneously measures changes in the resonance frequency (Δ*f*) and energy dissipation (Δ*D*) when the mass of an oscillating piezoelectric crystal changes upon increase/decrease in the mass of the crystal surface due to removal/deposition of the material. Dissipation refers to the frictional losses that lead to damping of the oscillation depending on the viscoelastic properties of the material. For a rigid adsorbed layer that is fully coupled to the oscillation of the crystal, Δ*f*_*n*_ is given using the Sauerbrey equation^29^ (eq 1)
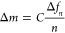
1where Δ*f*_*n*_ is the observed frequency shift, *C* is the Sauerbrey constant (−0.177 mg Hz^–1^ m^–2^ for a 5 MHz crystal), *n* is the overtone number (*n* = 1, 3, 5, etc*.*,), and Δ*m* is the change in mass of the crystal due to the adsorbed layer. The mass of a soft (i.e., viscoelastic) film is not fully coupled to the oscillation, and the Sauerbrey relation is not valid because energy is dissipated in the film during the oscillation. The damping (or dissipation) (*D*) is defined as (eq 2)
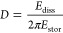
2where *E*_diss_ is the energy dissipated and *E*_stor_ is the total energy stored in the oscillator during one oscillation cycle.

#### 2.7.1 H_2_O/D_2_O Exchange Studies

The water content of thin PDMS films before and after coating with Chi-77KS and Chi-77KS/AMOX was determined using a H_2_O/D_2_O exchange as described previously.^30−32^ The coated crystals were placed in the QCM-D flow cell and equilibrated with ultrapure water until a stable frequency was obtained (*t* ∼ 120 min). Subsequently, the experiments were restarted and a baseline in ultrapure water was set up for 10 min. After this step, ultrapure water was exchanged with D_2_O for 15 min. Then, D_2_O was exchanged with ultrapure water for 15 min. Afterward, the experiments were ended.

#### 2.7.2 Adsorption of Model Blood Proteins

The Chi-77KS and Chi-77KS/AMOX-coated PDMS Au-crystals were mounted in the QCM-D flow cell and equilibrated with ultrapure water, and subsequently with PBS until a stable change in frequency was established. The BSA, FIB, GLO, or their mixture was pumped through the QCM-D cell for 60 min, followed by rinsing with PBS solution for 60 min at the constant flow rate *Q* = 0.1 mL min^–1^. Adsorption experiments were performed in triplicates at pH 7.4 and 37 ± 0.1 °C to simulate the physiological conditions in the human body. Experiments on protein adsorption (BSA, FIB, GLO, or their mixture) were also performed on the PEG-thiol (PEG-SH)-coated surfaces (negative control) under the same conditions as described above. PEG-SH (1 mg mL^–1^, dissolved in PBS buffer, pH 7.4) was pumped onto clean Au-crystals for 30 min after the crystals were equilibrated with PBS for 30 min. The coated PEG-SH layers were rinsed with PBS for 30 min. The solutions were pumped at a flow rate of 0.1 mL min^–1^ and at 21 ± 0.1 °C.

##### 2.7.2.1 Viscoelastic Modeling

The viscoelastic Voigt model was applied for calculating the adsorbed mass (Γ_QCM_), film thickness (*h*_f_), viscosity (η_f_), and elastic shear modulus (μ_f_) of the adsorbed protein layers. In this model, the adsorbed layer was treated as a viscoelastic layer between the quartz crystal and a semi-infinite Newtonian liquid layer. More details on the Voigt modeling can be found elsewhere.^33,34^ For data evaluation or fitting, the different overtones (*n* = 3, 5, 7, 9, and 11) of frequency and dissipation were used. All calculations were carried out using the software package QTools 3.0.12 (Q-Sense, Biolin Scientific, Gothenburg, Sweden). The fitting parameters used in the modeling are: viscosity, from 1 × 10^–4^ to 0.01 N s m^–2^; elastic shear modulus, from 1 × 10^4^ to 1 × 10^8^ N m^–2^; and thickness, from 1 × 10^–10^ to 1 × 10^–6^ m. It is worth noting that the values of *h*_f_ and ρ_f_ were not independent variables. In order to calculate the effective thickness and adsorbed mass (eq 3), the density ρ_f_ values were varied between 1000 and 1180 kg m^–3^. It turned out that no mass change for the adsorbed layer was occurred by changing the density value, and therefore the density (ρ_f_) of 1000 kg m^–3^ was used for all calculation (eq 3)
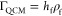
3

#### 2.7.3 Biofilm Formation Assay

Reduction of biofilm formation on coated PDMS disc was assessed relatively to the noncoated PDMS disc using *E. coli* ATCC 25922 as a representative of Gram-negative bacterium and *S. aureus* ATCC 29213 as a representative of Gram-positive bacterium. Bacteria were revitalized from −80 °C on TSA medium and precultured twice before a single colony was inoculated into 5 mL TSB and incubated at 37 °C and 180 rpm overnight. The inoculum was diluted with an equal volume of sterile 1× PBS of pH 7.4, and an aliquot of 50 μL was transferred into 200 mL baffled Erlenmeyer flasks containing 50 mL TSB and blank and coated PDMS discs. All discs were rinsed with 70% ethanol (v/v) and dried near the Bunsen burner before inserting into the flasks. The flasks were incubated at 37 °C and 180 rpm for 24 h. Both neat and functionalized PDMS discs were taken out of the flasks and rinsed with sterile 1× PBS to remove the planktonic bacteria that had attached to the disc surface. The discs were then placed separately into 15 mL centrifuge tubes with 3 mL of TSB and vortexed simultaneously (10 min at 2000 rpm) to dislodge and disrupt the biofilm from the surface. Following serial dilutions in 1× PBS (pH 7.4), 100 μL of each dilution was spread onto TSA in triplicates and incubated for 24 h at 37 °C to evaluate bacteria attached on the disc surface. The concentration of bacteria that remained attached on each treated (Chi-77KS, Chi-77KS/AMOX) and neat PDMS surface was expressed as CFU mL^–1^ (taking into account dilution factors) and compared regarding the significant differences.

### 2.8 Statistical Analysis

All numerical values are given as mean ± standard deviation (SD). Statistical analysis was performed using Prism 8.4.3 (GraphPad, San Diego, CA, USA). A one-way ANOVA (and nonparametric) followed by a Dunnett test, and the student’s *t*-tests (nonparametric) were carried out. The Student’s *t*-test was used for obtaining nonparametric data. *P*-values < 0.05 were considered statistically significant. Samples that show a significant difference compared to the control sample are marked with * for ANOVA and # for Student’s *t*-test.

## 3 Results and Discussion

### 3.1 Functionalization of “Model” PDMS Films with Nanolayers of Chi-77KS and Chi-77KS/AMOX Complex

**Figure 1:**
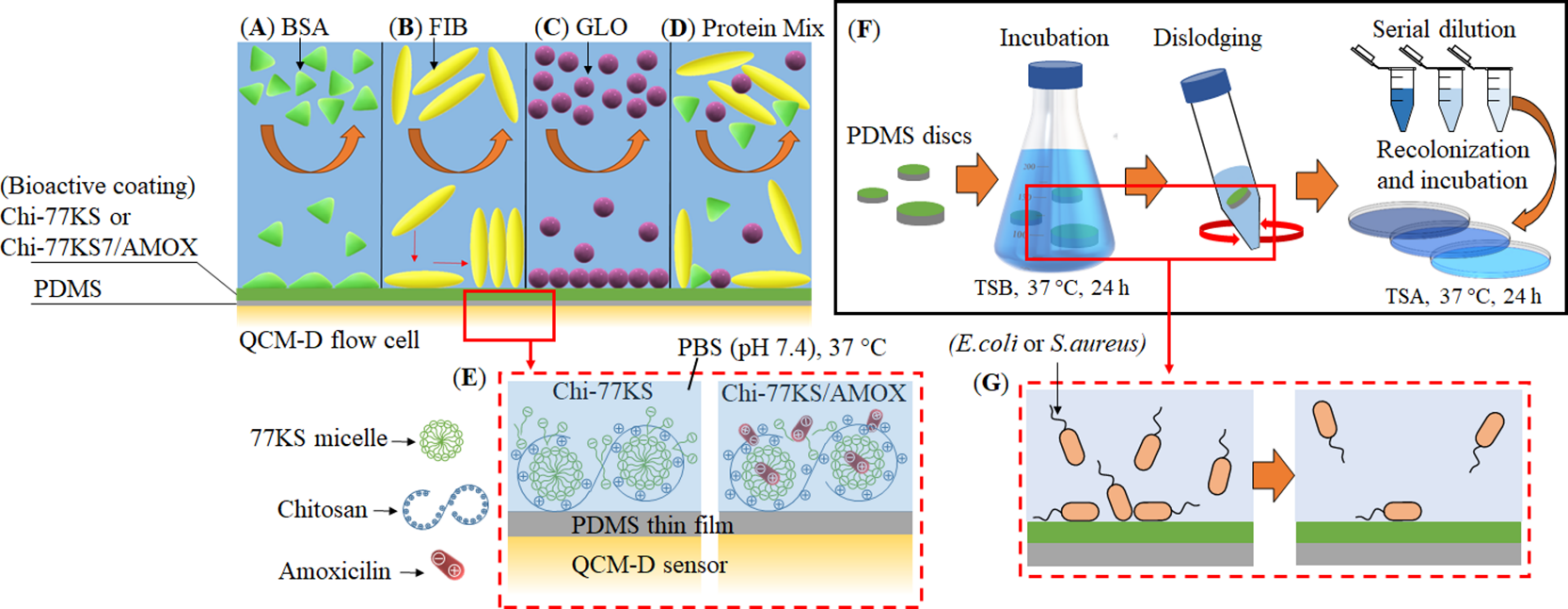
Illustration of design and functionalization of PDMS implants with bioactive coatings of Chi-77KS and Chi-77KS/AMOX. Adsorption of BSA (A), FIB (B), GLO (C) and their mixture (D) on bioactive nanolayers (Chi-77KS and Chi-77KS/AMOX) coated on “model” PDMS surfaces at pH 6.5 and at ambient temperature, (E) presence of different functional molecules (77KS anionic surfactant in the micelle form, cationic chitosan, and amoxicillin drug) in the bioactive nanolayers. Biofilm formation assay preparation with bacteria (*E. coli* and *S. aureus*) (F) and their interaction (G) with “real” PDMS discs functionalized with and without bioactive coatings at pH 7.4 and 37 °C.

Despite their numerous applications in biomedicine (e.g., in catheters, prosthetics, and stent development), the main disadvantages of PDMS-based biomaterials are their high hydrophobicity and lack of active functional sites, resulting in unspecific adsorption of proteins and biofilm formation in contact with body fluids. To overcome this, we functionalized the neat PDMS surfaces with a PESC (PESC: Chi-77KS) consisting of a natural polymer Chi (cationic) and lysine-based surfactant 77KS (anionic). In addition, we incorporated AMOX, an antibiotic known to reduce the bacterial infection, into the PESC (Chi-77KS/AMOX) to enhance the antimicrobial properties or bioactivity of the final coatings. These developed synergistic PESCs with and without incorporated AMOX were adsorbed onto “model” PDMS surfaces using QCM-D^24^ (Figure 1E).

**Figure 2:**
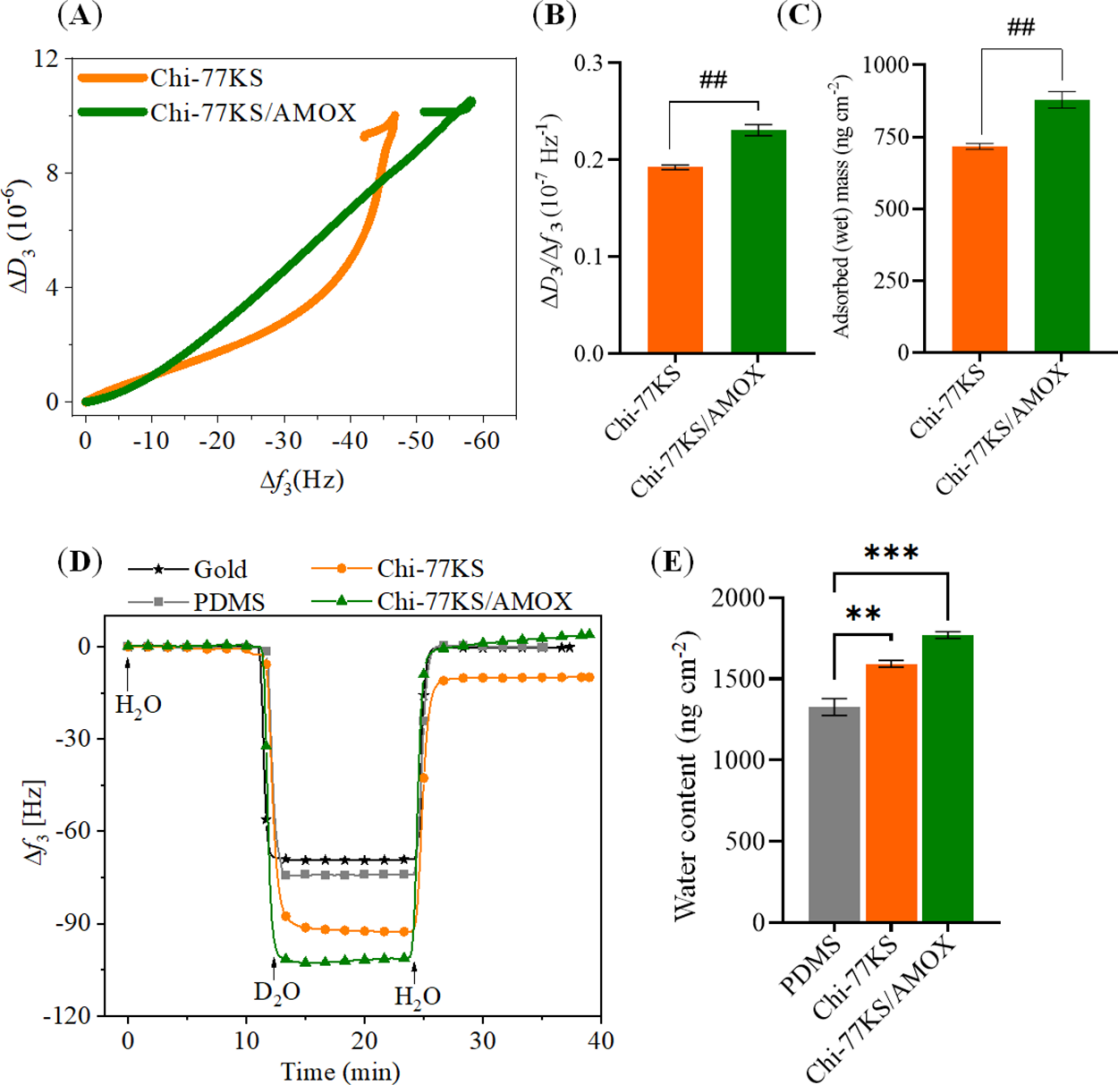
(A) QCM-D change in dissipation versus frequency, (B) ratio of Δ*D*_3_/Δ*f*_3_, (C) wet mass—data analysis was done by Student’s *t*-test with Dunnett test, values are presented as ± SD; ##*p* < 0.05, (D) H_2_O/D_2_O exchange, and (E) water content for the Chi-77KS and Chi-77KS/AMOX complex adsorbed onto “model” PDMS surfaces; data analysis was done by one-ANOVA with the Dunnett test, values are presented as ± SD; **p < 0.05, ***p < 0.05 (compared to negative control PDMS).

The slope of the dissipation change (Δ*D*_3_) versus the frequency change (Δ*f*_3_) shown in Figure 2A reflects the viscoelastic properties of the adsorbed layers of the Chi-77KS and Chi-77KS/AMOX complex at 25 °C and at pH 6.5. The slope of the curve can be correlated with the viscoelastic properties of the adsorbed layers. The higher slope indicates a more viscous or hydrated layer. Compared to Chi-77KS (without AMOX), the adsorption of Chi-77KS/AMOX showed a more loosely packed and strongly hydrated layers, which is reflected by a higher slope. This can be further confirmed by a maximum Δ*D*_3_/Δ*f*_3_ ratio observed for Chi-77KS/AMOX (Figure 2B), meaning that the adsorbed layer with a high Δ*D*_3_/Δ*f*_3_ ratio is considered highly viscoelastic, that is, hydrated.^31,35^ Such hydrated layers have enormous potential to control unspecific adsorption of proteins or microbes at the interfaces of PDMS-based medical implants. Interestingly, in comparison to Chi-77KS/AMOX, the curve of Chi-77KS during adsorption is noticeable, implying different adsorption kinetics. Although the change in the dissipation value at the end of desorption is nearly the same for both systems, a low change in dissipation from the beginning to half of the adsorption, followed by a sudden increase in dissipation at the end of the adsorption is observed for Chi-77KS compared to the same PESC but incorporated with AMOX. This indicates that the adsorption progressed rapidly in the initial phase and formed a tightly (rigidly) bound layer (*ca.* 41 nm) with less incorporated water, followed by the formation of a more swollen or hydrated top layer as the adsorption continued.^14,31,35,36^ On the contrary, for Chi-77KS/AMOX, a steady increase in dissipation and a steep slope are observed, suggesting the formation of more loosely adsorbed layers (*ca.* 61 nm) on the PDMS surface with more incorporated water. For both systems, however, most of the adsorbed mass remained on the PDMS surface after rinsing with water (Figure 2C, Chi-77KS: 717 ± 18 ng cm^–2^, Chi-77KS/AMOX: 879 ± 50 ng cm^–2^), indicating the irreversible and strong binding of the PESC to the hydrophobic PDMS surface. The strong binding between the neat PDMS and Chi-77KS/AMOX is assumed to be due to physical interactions, including H-bonds, van der Waals, and hydrophobic interactions.^24^ The latter could play a major role due to the presence of long hydrophobic linear alkyl chains and aromatic moieties in 77KS and AMOX. In addition, the desorption curves do not overlap with the adsorption curves. This indicates that the viscoelastic properties of the adsorbed layers are altered, due to conformational changes or rearrangement of the molecules adsorbed on the surface during rinsing. Overall, we could show that the multifunctional nanolayers on PDMS implant surfaces can be created by our synergistic PESCs (Chi-77KS and Chi-77KS/AMOX) without the need of any harsh physical or chemical treatments.

To quantify the water content of the neat “model” PDMS films before and after functionalization with nanolayers of Chi-77KS and Chi-77KS/AMOX, we performed H_2_O/D_2_O exchange studies using QCM-D (see Figure 2D) and the calculated water content of these layers is shown in Figure 2E. The differences in Δ*f*_3_ and the water content between the neat PDMS films and the later film functionalized with nanolayers of Chi-77KS and Chi-77KS/AMOX are evident. Neat PDMS alone exhibited Δ*f*_3_ of −74 ± 2 Hz (1310 ± 40 ng cm^–2^), which increased to −92 ± 1.7 Hz (1628 ± 55 ng cm^–2^) and −102 ± 2 Hz (1810 ng cm^–2^) for Chi-77KS and Chi-77KS/AMOX coated layers, respectively. This indicates that the water content increased to 25 and 40% for Chi-77KS and Chi-77KS/AMOX surfaces, respectively, and the functionalized surfaces became more hydrophilic compared to the neat and hydrophobic PDMS. The determined water content agrees well with the adsorbed mass (Figure 2C) and the ratio of Δ*D*_3_/Δ*f*_3_ (Figure 2B, related to surface hydration), the latter being higher for Chi-77KS/AMOX. The significant increase in the water content can be related to the hydration nature of the hydrophilic and charged groups (e.g.: −NH_2_, −COOH and −OH) present in the functionalized layers of both Chi-77KS and Chi-77KS/AMOX. Such coated functional layers on the hydrophobic PDMS with a higher water content or hydration capacity are beneficial to prevent the unspecific adsorption of proteins.^30,31,37^

### 3.2 Morphology and Wettability of Functionalized “Model” PDMS Surfaces

**Figure 3:**
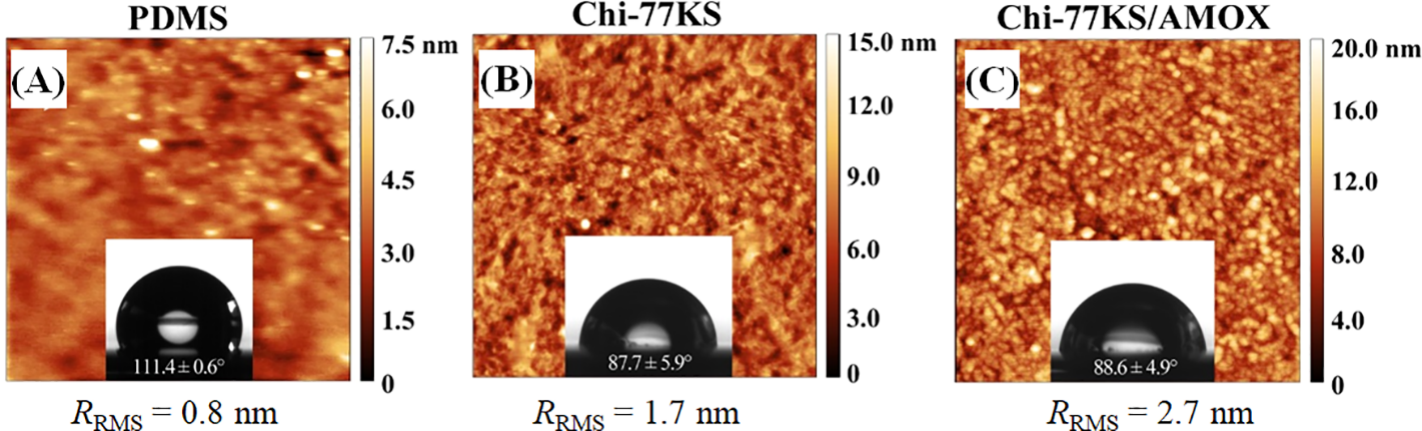
AFM height images and water contact angles of neat (A), Chi-77KS- (B), and Chi-77KS/AMOX-coated PDMS surfaces (C). Image size: 2.5 × 2.5 μm^2^.

The AFM height images (Figure 3) show the PDMS “model” surface prior to and after coating with Chi-77KS and the Chi-77KS/AMOX complex. The height image of neat PDMS shows a relatively homogeneous and smooth surface with *R*_rms_ = 0.8 nm. On the contrary, both Chi-77KS (Figure 3B) and Chi-77KS/AMOX (Figure 3C)-coated surfaces show increased *R*_rms_ with 1.7 and 2.7 nm, respectively. Interestingly, Chi-77KS/AMOX appears to have higher *R*_rms_ and slightly different distribution of the applied materials, compared to Chi-77KS, which might result from the difference in surface chemical composition due to the AMOX-loaded complex and its structures (micelles). Chi-77KS is loaded by AMOX inside and possibly carries it at the external, or even both parts of the complex (see Figure 1E). The neat PDMS surface is completely and uniformly covered by the coating of both Chi-77KS and the Chi-77KS/AMOX complex (Figure 3). However, higher roughness and thickness indirectly proved successful adsorption of both PESCs on the PDMS without requiring any special or tedious surface treatment. As expected, the hydrophobic neat PDMS surface showed a low wettability with SCA(H_2_O) value of (111.4 ± 0.6)°. This decreased to SCA(H_2_O) values of (87.7 ± 5.9)° and (88.6 ± 4.9)° after coating with Chi-77KS and Chi-77KS/AMOX complex, respectively. This shows that the adsorbed nanolayers substantially reduced the hydrophobicity of PDMS due to the introduction of polar and charged groups.^14,15,31^ However, the obtained SCA(H_2_O) of the two functionalized samples are on the edge of hydrophobicity and appear more hydrophilic.

### 3.3 Zeta potential of Neat and Functionalized “Model” PDMS Surfaces

**Figure 4:**
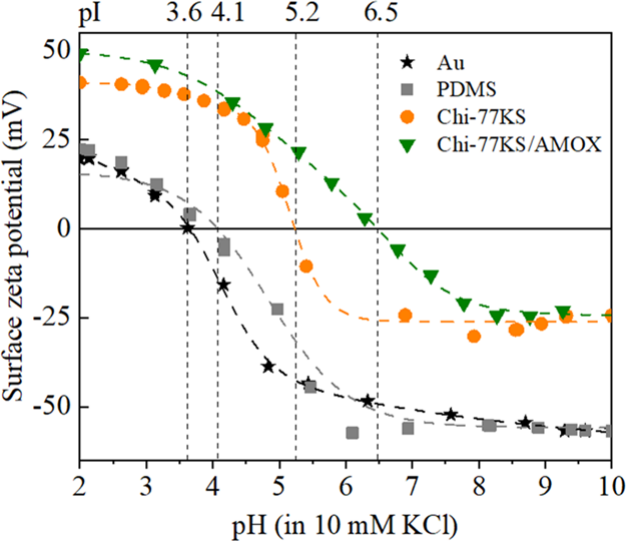
Surface ζ-potential of neat surfaces of Au and PDMS, and PDMS surfaces coated with Chi-77KS and Chi-77KS/AMOX nanolayers.

The surface ζ-potential was measured to observe the charging behavior of Chi-77KS, and Chi-77KS/AMOX adsorbed onto PDMS “model” surfaces (Figure 4). It is an essential tool to study the charging behavior, isoelectric point (pI), and hydrophilicity/hydrophobicity of samples in aqueous solution; all of them can be followed simultaneously before and after treatment. Recently, the streaming potential method (to determine electrokinetic or ζ-potential) has been largely used to study the influence of coating onto surface-charging behavior.^38^ As is shown in Figure 4, the bare Au surface of QCM-D crystals exhibited negative ζ-potential above the pI = 3.6, which is in agreement with the value reported in the literature.^28^ In comparison, the observed pI = 4.1 for PDMS is higher than that of the Au-surface, and such pI is generally found for hydrophobic surfaces that do not possess surface functional groups.^39^ For instance, the neat PDMS displayed negative ζ-potential values (−52 mV at pH 7.4) at a pH higher than 4.1.^35^ The observed negative ζ-potential values for both neat Au and PDMS can be explained by the specific adsorption of water ions at the interfaces of the latter materials as reported by other authors.^38,40,41^ Compared to neat PDMS, the Chi-77KS and Chi-77KS/AMOX functionalized PDMS surfaces carry mixed functional groups (e.g.*,* −NH_2_, −COOH, and −OH) and thus due to their protonation/deprotonation, differences in the pI and ζ-potential are to be expected. As such, higher pI = 5.2 and positive ζ-potential values at pH > 5.2 are observed for the Chi-77KS-coated surfaces than the neat PDMS (pI = 4.1), indicating that the presence of positively charged amino groups of Chi and a sufficient covering of the surface. Above this pH, a negative ζ-potential (a lower than the neat PDMS) value is detected. This is assumed to be due to the presence of anionic 77KS surfactant with −COO^–^ groups on the coated surface. Furthermore, the absolute values of ζ-potential increased positively at lower pH values due to Chi amino groups (−NH_2_), which protonate (−NH_3_^+^) in the acidic area below pH 6.3–6.5 (pI of neat Chi).^42^ For Chi-77KS/AMOX-coated surfaces, the shift of pI toward more alkaline from pI = 5.2 to pI = 6.5 is visible, which is assumed to be due to the positively charged secondary and primary groups of AMOX in/at formed PESC (p*K* of secondary amine: 7.5 and primary amine: 9.9, according to potentiometric titration of pure AMOX, see Figure S1A). A maximum positive ζ-potential and shifting of pI more to the theoretical value of Chi is observed compared to the coating of Chi-77KS. This suggests that the adsorbed complex on the PDMS surfaces is less covered or dominated by the negatively charged 77KS molecules as in the case of Chi-77KS-functionalized surfaces. It is expected that the carboxyl groups additionally present in AMOX molecules can contribute to a further increase of negative ζ-potential (pH > pI) for the Chi-77KS/AMOX surfaces compared to the same type of surfaces without AMOX. However, no increase in the negative ζ-potential is observed. It is suggested that electrostatic interactions between the protonated amino groups of chitosan (or amino groups of AMOX) and negatively charged carboxylic groups of AMOX in the PESC can occur. This can result in the blocking of carboxyl groups of AMOX. Therefore, the presence of such groups cannot be accessed during ζ-potential measurements. The higher reduction of ζ-potential to a less-negative value after Chi-77KS and Chi-77KS/AMOX adsorption may also be due to the reduction of hydrophobicity (differences in plateau regions) due to the introduction of polar groups, which is in accordance with the measured SCA(H_2_O) of the neat and functionalized PDMS surfaces (see Section 3.2).

### 3.4 Insights into the Adsorption of Serum Proteins

Although the hydrophobic PDMS-based medical implants are the key choice of biomaterials for several clinical applications, the biofilm formation on the implant surface due to spontaneous unspecific adsorption of blood proteins after (in vivo) implantation prevent their long-term safe usage. While the several functions of proteins such as serum albumin, FIB, and GLO are known in the human body, these charged proteins adsorb unspecifically on the implanted PDMS surfaces under the physiological conditions (i.e.*,* at pH 7.4 and 37 °C). Besides the current development of several new hydrophilic or charged functional materials to address the problems associated with the unspecific protein adsorption, the adsorption behavior or mechanism (e.g.*,* adsorption kinetics, conformation, and viscoelastic behavior) of single proteins (BSA, FIB, and GLO) and their mixture (competitive interactions) at the interfaces of PDMS-based biomaterial decorated with PESCs as in this current study remains poorly studied and understood. These multicharged surfaces prepared from the PESC nanocoating are rather natural-based, and can therefore be considered as an excellent bioactive coating for preventing unspecific protein or bacterial adsorption. In this study, we investigated the adsorption experiments with a lower concentration (1 mg mL^–1^) rather than mimicking the concentration of proteins found in the blood in order to avoid the binding kinetics of proteins “drown” in bulk effects.

#### 3.4.1 Adsorption of BSA

**Figure 5:**
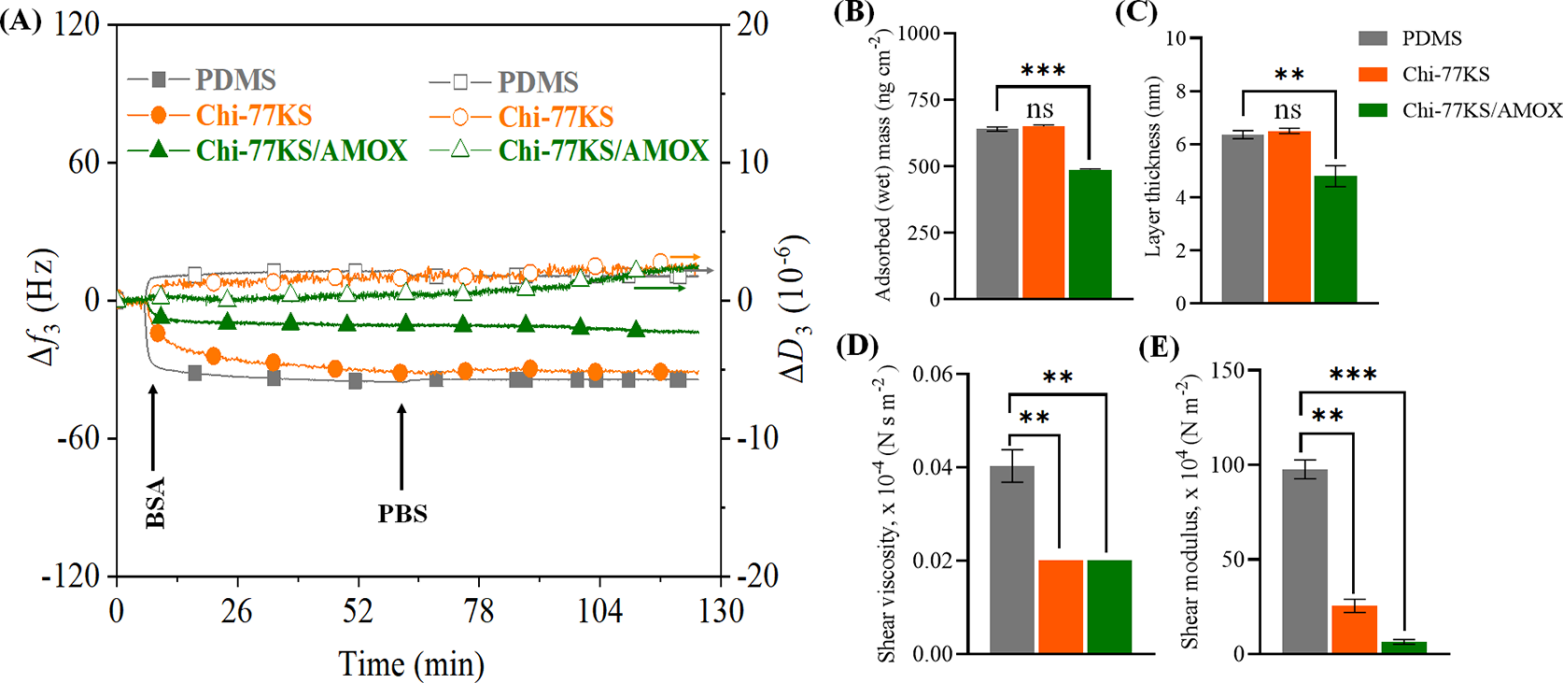
(A) QCM-D frequency (Δ*f*_3_) and dissipation (Δ*D*_3_) changes for the adsorption of BSA (at *c* = 1.0 mg mL^–1^ in PBS buffer, pH 7.4, *Q* = 0.1 mL min^–1^) on the neat and functionalized “model” PDMS surfaces and viscoelastic properties: (B) mass, (C) thickness, (D) shear viscosity, and (E) elastic shear modulus of the adsorbed BSA layer analyzed using the Voigt model; data analysis was done by one-ANOVA with the Dunnett test, values are presented as ± SD; **p < 0.05, ***p < 0.05 (compared to negative control PDMS), and nonsignificant (ns).

Proteins, in general, exhibit higher surface activity closer to their pI (net charge is zero), due to reduced electrostatic repulsion between uncharged adsorbing molecules (more molecules to bind) and altered protein structure/conformation (changes in charge of amino acids).^43^ In this study, we first investigated the interaction and unspecific protein adsorption nature of “model” PDMS surfaces coated with Chi-77KS and Chi-77KS/AMOX using BSA as a generally accepted marker, and whose adsorption is, in principle, driven mainly by hydrophobic interactions with the surface.^14,15,35^ Albumins are a group of proteins, occurring in the body fluids and tissues of mammals. Serum albumin is the most abundant protein in blood.^44^ BSA is amphoteric because it carries negatively charged amino acid groups (aspartic acid and glutamic acid) and positively charged lysine or histidine moieties.^45^ BSA has a pI at 4.9, while the solution (1 mg mL^–1^) is negatively charged at pH = 7.4 (adsorption condition), due to abundant and negatively charged carboxyl groups (−0.26 mmol g^–1^, Figure S1, Table S1). To evaluate the protein-repellent performance of nanocoatings of Chi-77KS and Chi-77KS/AMOX on PDMS, we compared the protein adsorption results of the later coatings with negative and positive control samples. The neat (uncoated) PDMS was used as a negative control, while the PEG-coated surface was used as a positive control. The latter was obtained by coating PEG-SH (1 mg mL^–1^, dissolved in PBS buffer) on the neat Au surface using QCM-D at pH 7.4. This resulted in a stable coating of PEG-SH with Δ*f*_3_ of −20 ± 2 Hz (354 ± 40 ng cm^–2^, see Figure S2) and SCA(H_2_O) of 31 ± 2° (SCA(H_2_O) of Au: 75 ± 1.3°). The QCM-D results (Figure 5A) show strong and almost irreversible adsorption of BSA on the neat PDMS surface (negative control), with a final frequency shift of −35.5 Hz, without a significant change after the rinsing step. These results are in good agreement with previously reported values.^35^

BSA adsorption on the AMOX-loaded coating (Chi-77KS/AMOX) with Δ*f*_3_ = −10.8 Hz before the rinsing step shows enhanced BSA-repelling behavior in contrast to a nonloaded coating (Chi-77KS) with Δ*f*_3_ = −31.3 Hz before the rinsing step, showing higher adsorbed protein mass. The rinsing step did not cause significant adsorption stability, as Δ*f*_3_ of Chi-77KS increased by 0.5 Hz, and Δ*f*_3_ of Chi-77KS/AMOX decreased by 2.7 Hz. This slight decrease in frequency indicates that the adsorbed BSA layer is swollen due to the incorporation of water molecules during rinsing with PBS buffer.^46^ Rinsing with PBS did not desorb considerable amounts of BSA, showing irreversible protein binding. Nevertheless, Chi-77KS and Chi-77KS/AMOX with Δ*f*_3_ of −30.8 and −13.5 Hz after the rinsing step, respectively, showed more than twofold improved protein-repellent behavior compared to neat PDMS with an equilibrium Δ*f*_3_ value of −34.3 Hz, which is still higher than the values obtained for PEG-SH surfaces (positive control, Δ*f*_3_: −5 ± 2 Hz, 89 ± 35 ng cm^–2^, Figure S3).

Dissipation data revealed more information about the hydration, rigidity, and viscoelastic properties of the adsorbed layer. With the same, but reversed trend, the Δ*D*_3_ values of PDMS, Chi-77KS, and Chi-77KS/AMOX showed constant increments, with notable relation with the adsorbed amount (Δ*f*_3_), as presented in Table 1. Higher negative frequency shifts for PDMS, Chi-77KS, and Chi-77KS/AMOX led to higher positive Δ*D*_3_ with 2.2, 1.7, and 0.4, respectively. Adsorption of BSA leads to a progressively softer layer, indicated with a positive Δ*D*_3_ shift, which keeps increasing, and settles after the PBS rinsing step due to the rearrangement of BSA molecules into a more packed formation, thus making the surface more rigid (lower Δ*D*_3_).^6^ A schematic of the proposed behavior of BSA protein adsorption is shown in Figure 1A.

**Table 1:** Frequency Change (Δ*f*_3_), Dissipation Change (Δ*D*_3_), Desorption Ratio Δ*f*_B_/Δ*f*_A_, and Δ*D*_3_/Δ*f*_3_ Ratio (A is Δ*f*_3_ and Δ*D*_3_ before Rinsing and B is the Final Δ*f*_3_ and Δ*D*_3_ after Rinsing) of the BSA-Adsorbed Layers

	Δ*f*_3_ [Hz]			Δ*D*_3_ [10^–6^]		Δ*D*_3_/Δ*f*_3_ [10^–7^ Hz^–1^]	
BSA	A	B	Δ*f*_B_/Δ*f*_A_ [%]	A	B	A	B
PDMS	–35.5 ± 2.0	–34.3 ± 1.7	96.6	2.2 ± 0.3	1.8 ± 0.4	0.6	0.5
Chi-77KS	–31.3 ± 1.7	–30.8 ± 3.0	98.4	1.7 ± 0.1	2.4 ± 0.8	0.5	0.8
Chi-77KS/AMOX	–10.8 ± 1.0	–13.5 ± 1.5	125.0	0.4 ± 0.1	1.7 ± 0.1	0.4	1.3

In general, the layer with high Δ*D*_3_/Δ*f*_3_ is considered as hydrated or viscous, which is profoundly favorable when designing a protein-repelling surface.^35^ Data in Table 1 reveal that higher Δ*D*_3_/Δ*f*_3_ follows reduced frequency change (adsorbed mass) and the BSA layer thickness relatively. The results of viscoelastic modeling (Figure 5B–E, Table S2) revealed the estimated BSA layer thicknesses (and mass) of 6.39 ± 1.04 nm (639 ± 8 ng m^–2^) for neat PDMS, 6.50 ± 0.98 nm (650 ± 5 ng m^–2^) for Chi-77KS, and 4.88 ± 0.35 nm (488 ± 2 ng m^–2^) for Chi-77KS/AMOX. The observed lower viscosity (*D*) and elastic shear modulus (*E*) of functionalized nanolayers are significantly different from that of the neat PDMS, indicating that both Chi-77KS and Chi-77KS/AMOX layers are incorporated with larger amounts of water or otherwise highly hydrated. Under this condition, the adsorbing proteins are not only repelled, but the remained adsorbed proteins are bound to the surface in the swollen and nonrigid conformation, resulting in low viscosity and elastic shear modulus of the adsorbed layers.

Besides the hydration forces, other interaction types should be considered as well. For example, BSA adsorbed on the coated surface, although the surface carries a negative net charge at pH 7.4 (ζ-potential results; Figure 4), it should repel BSA due to electrostatic repulsion. Weak repulsive forces cannot prevent proteins from approaching the coated surface and establishing physical and hydrophobic interactions (the DLVO theory).^47^ Hydrophobic interactions are the most pronounced for PDMS, which exhibits a hydrophobic character with a SCA(H_2_O) of 111.4 ± 0.6°. The adsorption is, thus, faster in the first few minutes on the neat PDMS compared to the functionalized surfaces. In contrast to the hydrophobic PDMS surface, BSA has a lower affinity toward hydrophilic ones. Chi-77KS and Chi-77KS/AMOX indicate more hydrophilic surfaces, with a SCA(H_2_O) of 87.7 ± 5.9° and 88.6 ± 4.9°, respectively, and lower protein adsorption. It is remarkable that the total density of the anionic charge on the adsorbate is too low for repulsion forces dominating over van der Waals forces. Surprisingly, the negative surface ζ-potential of Chi-77KS/AMOX is similar to that of Chi-77KS, although adsorption of BSA on Chi-77KS/AMOX is lower (Δ*f*_3_ = −10.8 Hz). This may be due to the complex and branched structure of the Chi-77KS/AMOX attached on the PDMS, which provides possible steric repulsive obstacles for the binding of proteins. From the results, it may be suggested that positive or negative electrostatic forces arising from a solid surface or BSA could not be a primary factor when determining the BSA adsorption behavior.^44^ In this case, the hydrophobic interactions, H-bonds, and van der Waals forces are the driving mechanism for the adsorption. BSA adsorption behavior suggests a single-step process due to its relative globular shape in comparison to FIB (Figure 5A). Thus, the adsorption of single molecules in any orientation occurs in approximately the same area coverage and packing. Further BSA adsorption is a competitive process to occupy free sites, which is being limited progressively as coverage increases.^6^

#### 3.4.2 Adsorption of Fibrinogen

**Figure 6:**
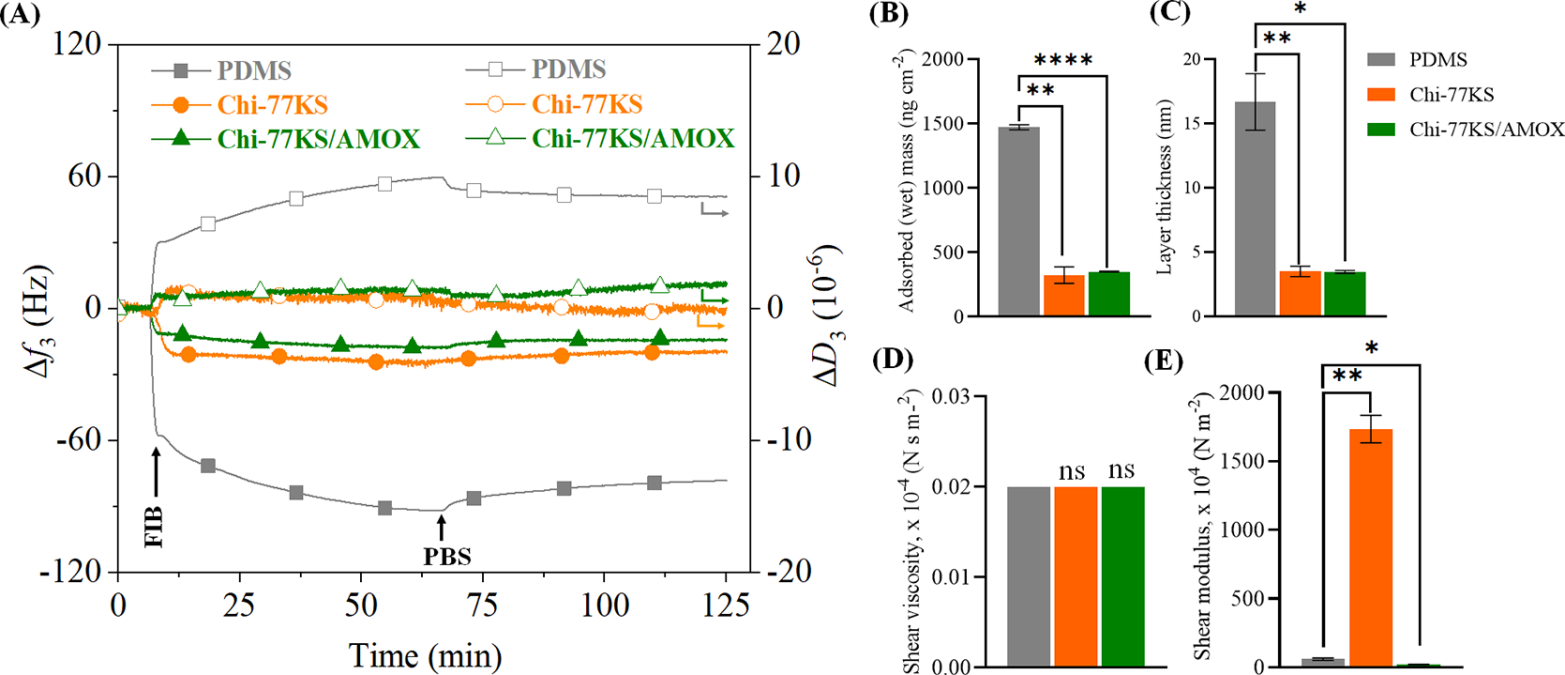
(A) QCM-D frequency (Δ*f*_3_) and dissipation (Δ*D*_3_) changes for the adsorption of FIB (at *c* = 1.0 mg mL^–1^ in PBS buffer, pH 7.4, *Q* = 0.1 mL min^–1^) on PDMS and bioactive coatings and viscoelastic properties: (B) mass, (C) thickness, (D) shear viscosity, and (E) elastic shear modulus of the adsorbed FIB layer calculated using the Voigt-based viscoelastic model; data analysis was done by one-ANOVA with the Dunnett test, values are presented as ± SD; *p < 0.05, **p < 0.05, ***p < 0.05 (compared to negative control PDMS), and nonsignificant (ns).

FIB has a central role in the activation of the blood coagulation cascade, fouling of artificial organs, platelet adhesion, thrombosis, leucocyte binding, and so forth.^48^ It is hydrophobic, large in size (340 kDa),^47^ and abundant with deprotonated carboxyl groups (−0.31 mmol g^–1^) at pH 7.4 (Figure S1C, Table S1).·In addition, FIB carries the least negative charge (−0.31 mmol g^–1^; BSA and GLO carry −0.26 mmol g^–1^ and −0.25 mmol g^–1^, respectively) and displays the most hydrophobic nature in comparison to BSA and GLO (pI = 4.3) as its pI is 5.8 (Figure S1, Table S1). In comparison with the adsorption of BSA (Δ*f*_3_ = −34.3 Hz) on the neat PDMS (negative control), FIB has a stronger affinity toward the hydrophobic surface with a final −78.3 Hz, which is 3-fold lower than the value observed for the positive control (Δ*f*_3_: −25 ± 1.3 Hz, 443 ± 23 ng cm^–2^, Figure S3). Chi-77KS/AMOX displayed the lowest frequency shift, with a maximum Δ*f*_3_ of −18.2 Hz and final Δ*f*_3_ of −14.3 Hz after the rinsing step (Figure 6A), although with a slight difference, Chi-77KS with Δ*f*_3_ of −25.1 Hz before and −20.2 Hz after rinsing with PBS. While these Δ*f*_3_ values are still lower compared to the positive control values, in the case of the Chi-77KS/AMOX coating, a twofold lower FIB adsorption is achieved, demonstrating the advantageous protein-repelling properties of our bioactive coating compared to the positive and negative controls. Interestingly, the final adsorbed FIB mass (Γ_QCM_) on the hydrophilic and functionalized surfaces of Chi-77KS and Chi-77KS/AMOX is found to be 355 ± 7 ng m^–2^ Hz and 349 ± 3 ng m^–2^, respectively, compared to 1468 ± 19 ng m^–2^ of the neat PDMS.

**Figure 7:**
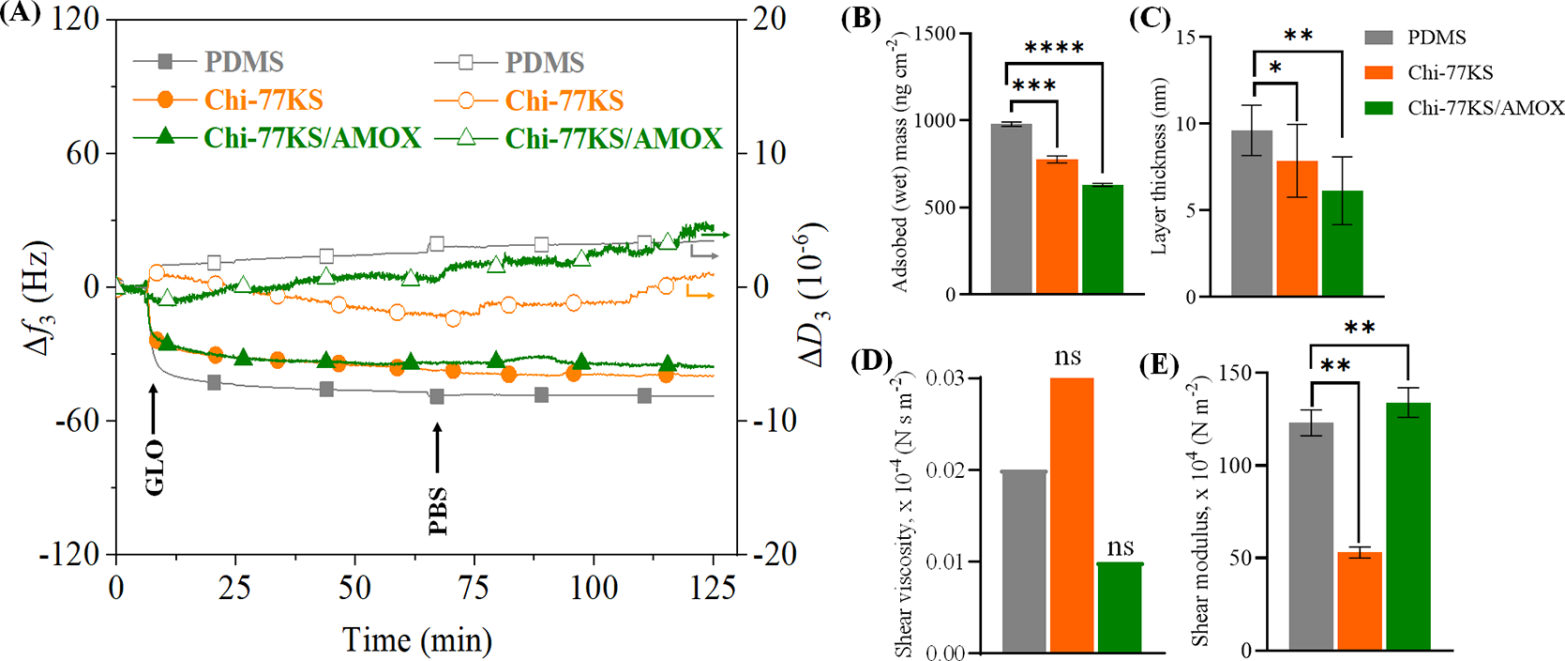
(A) QCM-D frequency (Δ*f*_3_) and dissipation (Δ*D*_3_) changes for the adsorption of GLO (at *c* = 1.0 mg mL^–1^ in PBS buffer, pH 7.4, *Q* = 0.1 mL min^–1^) on PDMS and bioactive coatings and viscoelastic properties: (B) mass, (C) thickness, (D) shear viscosity, and (E) elastic shear modulus of the adsorbed GLO layer calculated using the Voigt model; data analysis was done by one-ANOVA with the Dunnett test, values are presented as ± SD; *p < 0.05, **p < 0.05, ***p < 0.05, ****p < 0.05 (compared to negative control PDMS), and nonsignificant (ns).

PDMS showed the most significant Δ*D*_3_ change of 10.0 × 10^–6^ before and 8.5 × 10^–6^ after the rinsing step (Table 2, Figure 6A). Thus, it can be accepted that adsorbed FIB might pack closer to the surface to avoid water due to the hydrophobic (protein–water) forces prevailing over the electrostatic interactions (protein–protein). The Δ*D*_3_/Δ*f*_3_ data in Table 2 reveal that the FIB layer on Chi-77KS/AMOX is the most viscous/hydrated (1.3 × 10^–7^ Hz^–1^), while the Chi-77KS is the least viscous/hydrated (0.1 × 10^–7^ Hz^–1^). This might have resulted from FIB packing tightly to the surface, as observed through the Δ*D*_3_ change, with 1.0 × 10^–6^ before and −0.1 × 10^–6^ after the rinsing step. This can be further supported by the highest elastic shear modulus (Table S3) observed for the Chi-77KS/AMOX layer (μ_f_: 19 × 10^4^ N m^–2^) compared to the Chi-77KS (μ_f_: 1738 × 10^4^ N m^–2^) or to neat PDMS (μ_f_: 60 × 10^4^ N m^–2^). These huge differences in the elastic shear modulus can be related to the nature of FIB adsorption during which the size, shape, and interaction or binding within FIB and with functionalized layers have a strong impact on the viscoelastic properties of these adsorbed nanolayers^49^ (Figure 6D,E). Upon adsorption, the subsequent rinsing step leads to desorption of loosely bound FIB molecules and/or packing, due to the hydrophobic nature of FIB. Desorption of FIB from the surface is notable for all samples, indicating partially reversible adsorption or additional expelling of the water, caused by the change in FIB conformation on the surface. Apparently, the anionic carboxyl group on the surface repelled the carboxyl group of proteins and, thus, repulsive forces are also present to some extent. A multistage FIB adsorption proposal is presented schematically in Figure 1B. Upon binding, FIB molecules rearrange, and stack as proposed in Figure 1B, possibly due to intermolecular interaction, to achieve minimum energy and to reduce their contact with water. Because of the increased intermolecular hydrophobic interactions between the adsorbed FIB molecules, they may reorient by moving their long axis perpendicularly toward the surface.^6^ Thus, further molecules can adsorb on uncovered sites and maximize the adsorbed mass, which is the highest for the neat PDMS in comparison to the other two functionalized surfaces. The latter is also supported by the estimated wet thickness of the FIB layer, which was (14.68 ± 2.37) nm on the neat PDMS, (3.55 ± 0.25) nm on Chi-77KS, and was (3.49 ± 0.08) nm on Chi-77KS/AMOX (Figure 7C).

**Table 2:** Frequency Change (Δ*f*_3_), Dissipation Change (Δ*D*_3_), Desorption Ratio Δ*f*_B_/Δ*f*_A_, and Δ*D*_3_/Δ*f*_3_ Ratio (A is Δ*f*_3_ and Δ*D*_3_ before Rinsing and B is the Final Δ*f*_3_ and Δ*D*_3_ after Rinsing) of the FIB-Adsorbed Layers

	Δ*f*_3_ [Hz]			Δ*D*_3_ [10^–6^]		Δ*D*_3_/Δ*f*_3_ [10^–7^ Hz^–1^]	
FIB	A	B	Δ*f*_B_/Δ*f*_A_ [%]	A	B	A	B
PDMS	–92.1 ± 3.0	–78.3 ± 2.7	85.0	10.0 ± 1.3	8.5 ± 1.4	1.1	1.1
Chi-77KS	–25.1 ± 2.0	–20.2 ± 2.7	80.5	1.0 ± 0.2	–0.1 ± 0.01	0.4	0.1
Chi-77KS/AMOX	–18.2 ± 2.0	–14.3 ± 1.1	78.6	1.4 ± 0.3	1.9 ± 0.2	0.8	1.3

#### 3.4.3 Adsorption of γ-Globulin

GLOs are the dominant class of antibodies^50^ and the second most abundant plasma proteins after albumin. GLO may act as the most hydrophilic protein, with its lowest pI = 4.3 the farthest from the experimental pH 7.4 in comparison to BSA and FIB (Table S1). GLO is negatively charged at pH 7.4 (−0.25 mmol g^–1^; Figure S1D). The smallest differences in protein-repelling properties can be seen in the case of GLO, with the lowest difference between the neat PDMS with Δ*f*_3_ = −48.8 Hz and Chi-77KS (coating with the least FIB-repelling properties) with Δ*f*_3_ = −39.8 Hz being 9.0 Hz (Figure 7A). Among all bioactive coatings, Chi-77KS/AMOX exhibited the most improved protein-repellent properties, with −35.3 Hz after the rinsing step, which is 13.5 Hz higher Δ*f*_3_ than in the case of the neat PDMS, indicating fewer adsorbed GLO molecules. Even though the adsorbed mass of GLO on the coating of Chi-77KS is comparable to the value obtained for the positive control (Δ*f*_3_: −39 ± 3 Hz, 690 ± 53 ng cm^–2^, Figure S3), a 15% lower GLO adsorption is still observed for Chi-77KS/AMOX. In the initial phase, the adsorption profile of GLO on the neat PDMS exhibits a behavior similar to the one of BSA (Figure 5A), with rapid adsorption within the first 10–15 min (Figure 7A). The further negative Δ*f*_3_ and a decrease in the slope may indicate the reorganization of molecules to minimize the energy and to free up more sites, as already described previously.^51,52^ From the Δ*f*_3_ values, the adsorption behavior of GLO onto Chi-77KS displays initial rapid adsorption, with a subsequent decline over an extended period. A similar behavior in the case of Δ*f*_3_ is noticed for Chi-77KS/AMOX, but the dissipation showed the opposite trend, where it increased as the adsorption progressed.

Dissipation (Table 3, Figure 7A) shows that the surface of Chi-77KS becomes softer within the first minutes (initial rapid adsorption). However, the surface turns more rigid as Δ*f* begins to decline. Such a behavior may be due to adsorption of single GLO molecules to free sites in the first minutes. Afterward, the adsorbed molecules start to reorganize for a brief time, to allow more molecules to adsorb to free sites. Any further activities on the surface might include GLO undergoing conformational change and packing closer to the surface, due to the hydrophobic nature of the protein (Figure 1C). The rinsing step using PBS did not affect any mass changes (Δ*f*_3_), while the dissipation increased, this may be explained by the unfolding/unpacking of the protein. The adsorption of GLO onto a Chi-77KS/AMOX surface starts with rapid adsorption, while the surface becomes more rigid. Such a change may be possible due to the binding of GLO molecules to the surface, with accompanying expelling of water (possibly due to the more hydrated surface). As soon as the adsorption rate declines, the surface starts to become softer gradually over a more extended period. Upon rinsing, there was no significant frequency shift, while Δ*D*_3_ continued to increase, possibly due to protein unfolding/relaxation (by opening up its original structure). The Δ*D*_3_/Δ*f*_3_ ratios in Table 3 indicate that the GLO layer on Chi-77KS/AMOX is the most viscous/hydrated (1.3 × 10^–7^ Hz^–1^), while the Chi-77KS was the least viscous/hydrated (0.3 × 10^–7^ Hz^–1^). In contrast, the modeling results (Figure 7D,E, Table S4) showed a lower (μ_f_: 53 ± 3 × 10^4^ N m^–2^) and a higher (μ_f_: 134 ± 8 × 10^4^ N m^–2^) elastic shear modulus for the GLO-adsorbed surfaces of Chi-77KS and Chi-77KS/AMOX, which is somewhat surprising given the lower-level hydration for the former surfaces compared to latter ones according to the Δ*D*_3_/Δ*f*_3_ ratios. This can be related to different binding modes or reorganization of hydrophobic GLO on both surfaces with a particle-like morphology and roughness (see Section 3.2), which can have an impact on mechanical and viscoelastic properties of the adsorbed layers.^53^ The estimated layer thickness of the adsorbed GLO on the neat PDMS is 9.78 ± 1.56 nm, 7.76 ± 2.01 nm on Chi-77KS, and 6.29 ± 1.83 nm on Chi-77KS/AMOX (Figure 7C).

**Table 3:** Frequency Change (Δ*f*_3_), Dissipation Change (Δ*D*_3_), Desorption Ratio Δ*f*_B_/Δ*f*_A_, and Δ*D*_3_/Δ*f*_3_ Ratio (A is Δ*f*_3_ and Δ*D*_3_ before Rinsing and B is the Final Δ*f*_3_ and Δ*D*_3_ after Rinsing) of the GLO-Adsorbed Layers

	Δ*f*_3_ [Hz]			Δ*D*_3_ [10^–6^]		Δ*D*_3_/Δ*f*_3_ [10^–7^ Hz^–1^]	
GLO	A	B	Δ*f*_B_/Δ*f*_A_ [%]	A	B	A	B
PDMS	–46.9 ± 2.0	–48.8 ± 1.7	104.1	2.6 ± 0.7	3.5 ± 0.9	0.6	0.7
Chi-77KS	–38.6 ± 1.9	–39.8 ± 1.6	103.1	–2.3 ± 0.9	1.0 ± 0.1	0.6	0.3
Chi-77KS/AMOX	–33.8 ± 3.0	–35.3 ± 4.0	104.4	0.5 ± 0.1	4.5 ± 1.1	0.1	1.3

#### 3.4.4 Adsorption of the Mixed-Protein Solution

**Figure 8:**
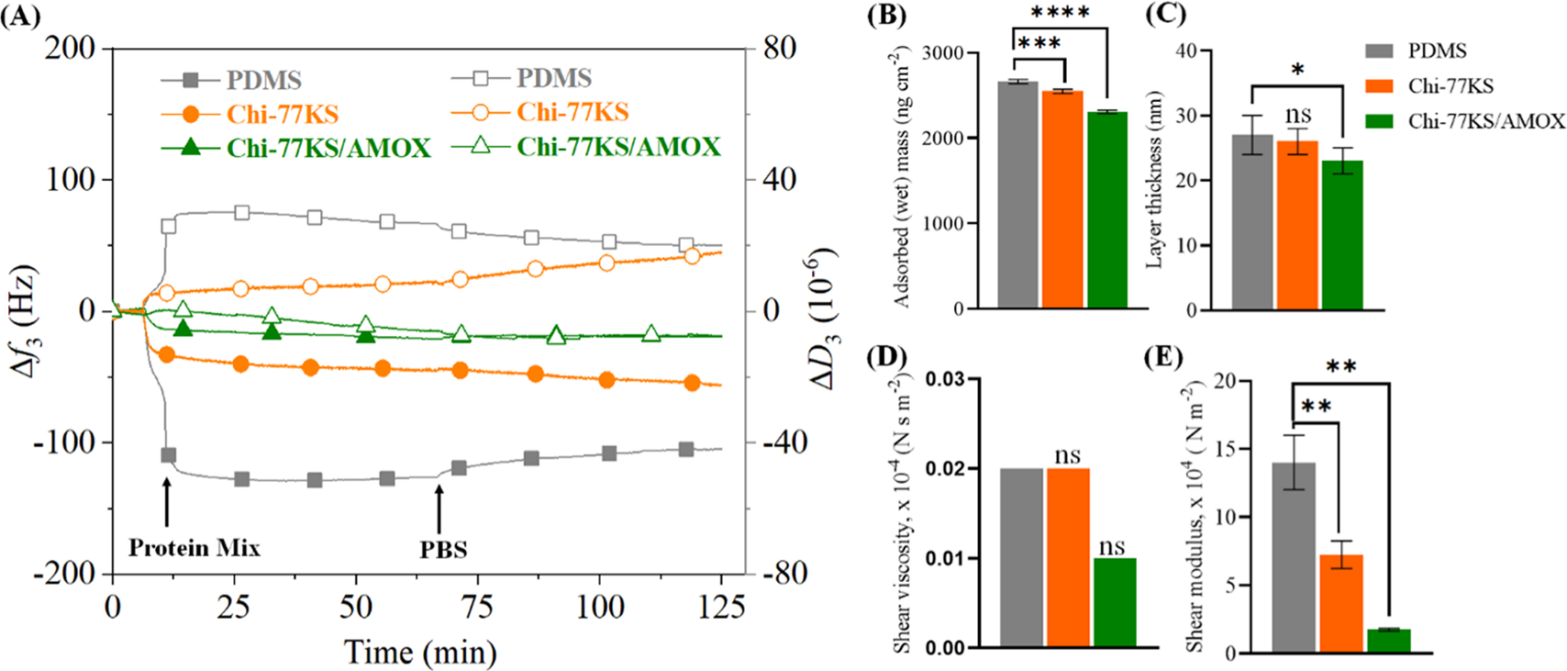
(A) QCM-D frequency (Δ*f*_3_) and dissipation (Δ*D*_3_) changes for the adsorption of mixed proteins (at *c* = 1.0 mg mL^–1^ in PBS buffer, pH 7.4, *Q* = 0.1 mL min^–1^) on PDMS and bioactive coatings and viscoelastic properties (B) Voigt mass, (C) thickness, (D) shear viscosity, and (E) elastic shear modulus of the adsorbed mixed protein layer calculated using the Voigt model; data analysis was done by one-ANOVA with the Dunnett test, values are presented as ± SD; *p < 0.05, **p < 0.05, ***p < 0.05, ****p < 0.05 (compared to negative control PDMS), and nonsignificant (ns).

In order to simulate and bring the process of protein adsorption closer to the “real” behavior with more accuracy, competitive adsorption was studied among proteins on different surfaces from mixed-protein solution. Speaking of competitive and sequential adsorptions, bulk protein concentration, protein–protein interactions, protein affinity to the surface, and change in conformation upon adsorption play a significant role.^44^ The concentrations of BSA, FIB, and GLO in the mixture for adsorption experiments were the same as for the single protein experiments (Section 2.2.3). The mixed protein solution obtained was clear and stable under the given conditions (without optically visible agglomeration).^36^ This could be because BSA could act as a dispersing agent, as it tends to reduce the intermolecular cohesion forces of the proteins, thus keeping them in the solution. From the results in Figure 8A, the general adsorption behavior of mixed proteins regarding the Δ*f*_3_ followed a similar pattern to that of FIB (Figure 6A), with considerable differences observed with the adsorbed amount on neat PDMS concerning all coated samples. Specifically, Δ*f*_3_ = −104.7 Hz of the neat PDMS is 48.3 Hz lower than that for Chi-77KS with Δ*f*_3_ = −56.4 (the lowest Δ*f*_3_ in comparison to all other cases, including Chi-77KS/AMOX and individual proteins). Interestingly, when comparing a single protein (Figures 5A, 6A and 7A) with the mixture (Figure 8A), the adsorbed amount of mixed-protein solution onto the neat PDMS is higher than for any other sample or single protein.

The adsorbed amount of mixed-protein solution on the Chi-77KS/AMOX with Δ*f*_3_ = −18.1 is lower than for Chi-77KS, with significantly higher Δ*f*_3_ = −56.4 Hz. There is a significant desorption behavior for the neat PDMS (Δ*f*_3_ = −126.2 to −104.7 Hz) after the rinsing step using PBS, while Chi-77KS/AMOX showed lower desorption, with an equilibrium value of −18.1 Hz (Table 4), which can be compared to the adsorbed amounts of mixed-proteins on the positive control (Δ*f*_3_: −18 ± 2 Hz, 319 ± 35 ng cm^–2^, Figure S3). All samples showed partially reversible protein adsorption. Chi-77KS behavior after the rinsing step can be explained with the unfolding of the proteins, as Δ*f*_3_ kept decreasing, from −45.1 Hz before and to −56.4 Hz after the rinsing step. In comparison, adsorption of the mixed-protein solution onto the neat PDMS resembled the adsorption behavior of FIB (Figure 6A), but with higher adsorbed mass (Δ*f*_3_). However, the adsorption of FIB on the neat PDMS surface is stronger, as Δ*f*_3_ = −78.3 Hz after and Δ*f*_3_ = −92.1 Hz before the rinsing step (15% desorption), or there might be more water expelled from the surface in the case of adsorption behavior of the mixed-protein solution on the neat PDMS surface. The difference before (Δ*f*_3_ = −126.2 Hz) and after (Δ*f*_3_ = −104.7 Hz) the rinsing step for the mixed-protein solution and neat PDMS is 17%.

**Table 4:** Frequency Change (Δ*f*_3_), Dissipation Change (Δ*D*_3_), Desorption Ratio Δ*f*_B_/Δ*f*_A_, and Δ*D*_3_/Δ*f*_3_ Ratio (A is Δ*f*_3_ and Δ*D*_3_ before Rinsing and B is the Final Δ*f*_3_ and Δ*D*_3_ after Rinsing) of the Mixed Protein-Adsorbed Layers

	Δ*f*_3_ [Hz]			Δ*D*_3_ [10^–6^]		Δ*D*_3_/Δ*f*_3_ [10^–7^ Hz^–1^]	
mixed-protein solution	A	B	Δ*f*_B_/Δ*f*_A_ [%]	A	B	A	B
PDMS	–126.2 ± 4.0	–104.7 ± 6.2	83.0	26.7 ± 2.1	20.1 ± 1.3	2.1	1.9
Chi-77KS	–45.1 ± 3.4	–56.4 ± 3.3	125.1	8.3 ± 2.0	18.1 ± 1.1	1.8	3.2
Chi-77KS/AMOX	–22.7 ± 2.2	–18.1 ± 1.4	79.7	4.8 ± 1.2	7.1 ± 0.9	2.1	3.9

Furthermore, the difference in dissipation lowered more rapidly after the rinsing step than for the adsorption behavior of FIB onto neat PDMS. Adsorption of the mixed-protein solution onto Chi-77KS followed a similar pattern to that of GLO (Figure 7A), with a slight difference regarding the Δ*D*_3_ behavior. There is a subsequent decline in dissipation upon rapid adsorption, with the surface turning to more rigid. In contrast, dissipation increased in a shorter period than for the GLO adsorption onto Chi-77KS. Thus, GLO may have the highest affinity toward adsorption on the Chi-77KS surface regarding its apparent competitive advantage over BSA and FIB. The data in Table 4 reveal that higher Δ*D*_3_/Δ*f*_3_ follows a reduced frequency change (lower adsorbed protein-mixture mass). The estimation of the adsorbed mass (and layer thickness) of the mixed-protein solution onto neat PDMS was 2662 ± 26 ng m^–2^ (27 ± 3 nm), on Chi-77KS was 2550 ± 21 ng m^–2^ (26 ± 2 nm), and on Chi-77KS/AMOX was 2308 ± 19 ng m^–2^ (23 ± 2 nm) (Figure 8A, Table S5). Interestingly, the observed Δ*f*_3_ is significantly lower compared to the Voigt mass for both functionalized surfaces, indicating the incorporation of larger amounts of water within the adsorbed layers of mixed proteins as predicted by the Voigt modeling. Adsorption of the mixed-protein solution onto Chi-77KS/AMOX indicated rapid initial adsorption with a subsequent decline. There may be notable desorption present after the rinsing step, although the dissipation behavior exhibited a brief lowering of Δ*D*_3_ as the surface turned more rigid. This may not be the case, as the difference probably originated from the fact that mixed-protein solution has a higher density and viscosity than PBS alone. Afterward, the surface continued to behave gradually more soft, which might be due to unfolding of the proteins adsorbed on the surface. This can be further confirmed by the modeling results, which showed that the Chi-77KS and Chi-77KS/AMOX layers with lower viscosity (Figure 8D) and elastic shear modulus (Figure 8E) are highly hydrated compared to the neat PDMS after protein adsorption. Compared to single protein adsorption, significantly lower viscosity and elastic shear modulus are observed for mixed-protein-coated surfaces; explains that the latter surfaces are highly hydrated or coupled with maximum water molecules, due to the presence of several polar and charged groups stemming from both adsorbing proteins and functionalized nanolayers. Generally, FIB might be the dominant protein in the mixture when adsorbing on the neat PDMS, while GLO might play a dominant role when the mixed-protein solution is adsorbed on Chi-77KS and Chi-77KS/AMOX.

#### 3.4.5 Comparison of Adsorption

BSA, FIB, and GLO were used for the adsorption on different bioactive functional coatings at pH 7.4 in PBS. At an experimental pH (7.4), BSA may not be as soluble as GLO, but more soluble than FIB, due to reduced charge repulsion based on its pI = 4.85 (Table S1). Additionally, FIB has the highest molecular weight and potentially more binding sites available. Generally, larger proteins interact stronger with the surfaces, as they are more surface-active due to their larger surface area and more surface-binding domains.^44^ In particular, increased protein–protein (hydrophobic) interactions are observed at pH values close to the pI, due to a lower solubility (reduced repulsion of charged protein groups).^54^ Protein–protein hydrophobic interactions overcome protein–water electrostatic interactions,^55^ and the adsorption maximizes closer to the pI. In such a way, proteins can form close-packed, dense (higher protein concentration), and, thus, a more viscous layer.^56^ Considering that the interaction of hydrophobic parts of the protein with water in an aqueous solution is energetically unfavorable, the protein molecule interacts with the surface to minimize the energy of the system. Adsorption on the hydrophobic surface is, thus, even more favorable, while a large positive entropy change occurs because of the exclusion of the water from the surface and the protein. The loss of water allows more interactions of proteins with the surface, while proteins undertake conformational change to maximize any surface interactions. Surface activity of the protein is influenced mostly by its primary structure; proteins are composed of amino acid submoieties, which, above p*K*, orient away from water, to minimize their interaction due to their hydrophobic (uncharged) nature.^57,58^ The driving force of hydrophobic interactions is the minimization of surface area/total interfacial free energy.^57^ Groups on the outside of the molecule that are commonly hydrophilic result in protein water solubility, although hydrophobic amino acids may also be available on the protein surface due to unfolding of the molecule to interact with the surface.^57,58^

**Figure 9:**
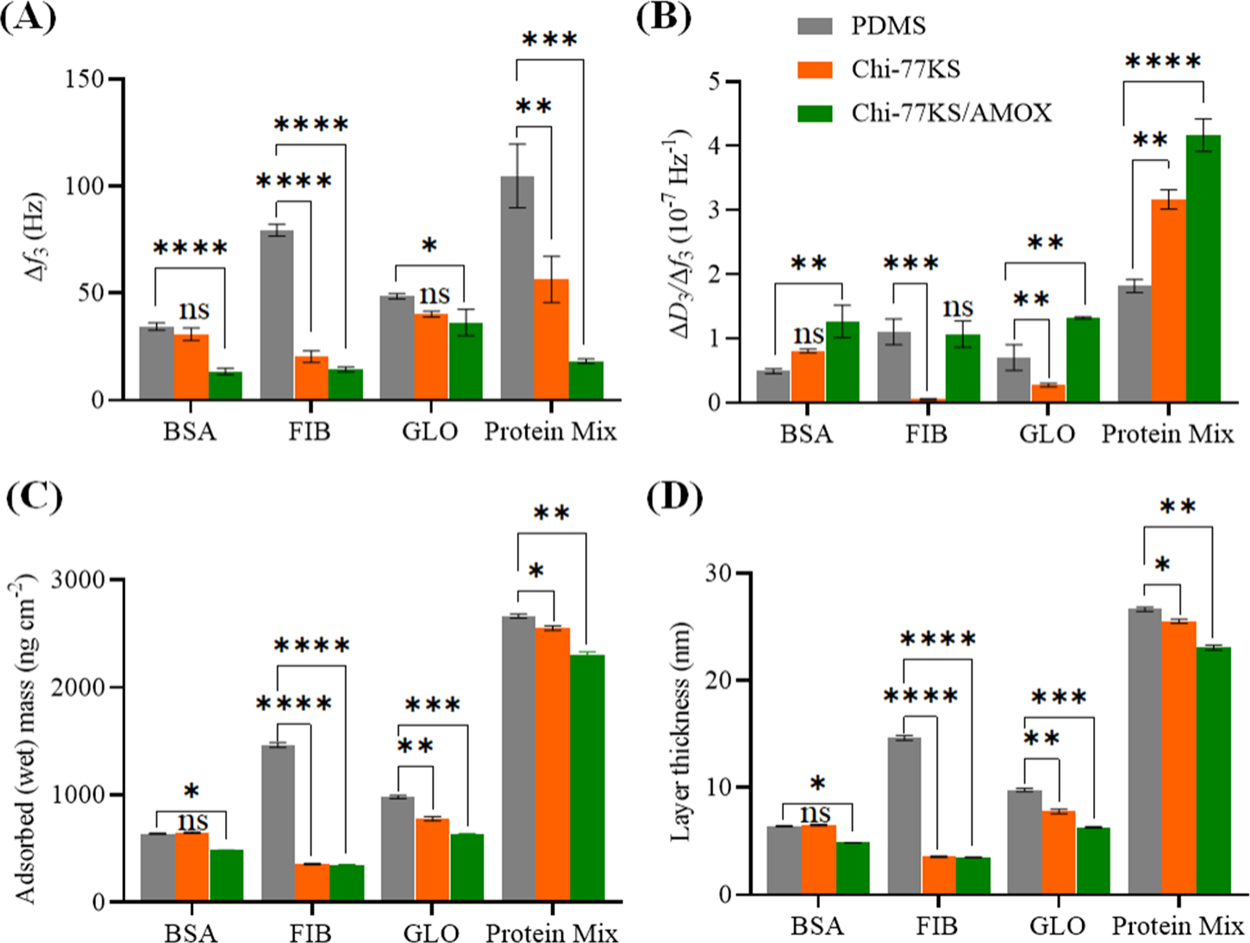
(A) QCM-D change in frequency, (B) ratio of Δ*D*_3_/Δ*f*_3_, (C) adsorbed viscoelastic (wet) mass, and thickness (D) from the combined display of BSA, FIB, GLO, and mixed-protein solution’s Δ*f*_3_ after the rinsing step for neat PDMS, Chi-77KS, and Chi-77KS/AMOX coated surfaces. Data analysis was done by one-ANOVA with the Dunnett test, values are presented as ± SD; *p < 0.05, **p < 0.05, ***p < 0.05, ****p < 0.05 (compared to negative control PDMS), and nonsignificant (ns).

Compared to the Chi-77KS and Chi-77KS/AMOX-functionalized surfaces, the proteins and their mixture have the highest affinity to adsorb to neat PDMS surfaces (Figure 9A,C), which are the most hydrophobic (SCA(H_2_O) = 111.4 ± 0.6°), have the lowest ζ-potential (−51.0 mV) and the lowest surface roughness (*R*_rms_ = 0.8 nm), and contain 25–40% less water. In general, the unmodified PDMS (negative control) showed enhanced unspecific interaction with both single and mixed protein solutions, resulting in a significantly increased adsorbed mass of proteins compared to PEG-SH (positive control). According to the QCM-D Δ*f*_3_ results, the following order of protein adsorption is observed for negative and positive controls: PDMS (protein-mix > FIB > GLO > BSA) and PEG-SH: (GLO > FIB > protein-mix > BSA). The Chi-77KS coating had the most hydrophilic surface, with SCA(H_2_O) = 87.7 ± 5.9° and lower ζ-potential (−30.0 mV), *R*_rms_ (1.7 nm), and 25% increased water content (1628 ± 55 ng cm^–2^). The Chi-77KS coating showed the evident improvement of protein-repellent behavior in the case of FIB and protein-mix, with significantly higher Δ*f*_3_ of −20.2 and −56.4 Hz, respectively. Compared to neat negative control PDMS, Chi-77KS-functionalized surfaces showed ∼74% higher Δ*f*_3_ for FIB and ∼46% higher Δ*f*_3_ for mix. With respect to the positive control, the same Chi-77KS surfaces exhibited slightly improved FIB repellency, similar repellency to GLO, but 15-fold and 3-fold higher repellence to BSA. The surface Chi-77KS/AMOX showed the most significant overall improvement of protein-repellent behavior (compared to both positive and negative control) and the highest ζ-potential (−15.7 mV), *R*_rms_ (2.7 nm), SCA(H_2_O) (88.6 ± 4.9°), and 40% more water content. Even though the repellence to BSA is not significant compared to the positive control, Chi-77KS/AMOX exhibited a twofold increase and similar repellence against FIB, GLO, and mixed protein solutions. Thus, it can be stated that functionalization of PDMS coatings with Chi-77KS and Chi-77KS/AMOX regarding protein-repelling behavior is relatively successful, with improved protein-repelling properties for both single and mixed-proteins samples. Overall, we demonstrated that PDMS functionalized with Chi-77KS/AMOX better prevents the adsorption of BSA compared to natural or synthetic hydrophilic coated surfaces [gelatin:^19^ 700 ng cm^–2^, polyvinylpyrrolidone (PVP):^20^ 2200 ng cm^–2^, chondroitin sulfate (CS):^21^ 500 ng cm^–2^, and carboxymethyl dextran:^59^ 300 ng cm^–2^]. Similarly, the FIB-repellent properties of Chi-77KS/AMOX as well as Chi-77KS functionalized surfaces are still comparable or even better than those of other hydrophilic surfaces reported in the literature, such as dendritic polyglycerol sulfate:^60^ 1900 ng cm^–2^, CS:^21^ 500 ng cm^–2^, PVP:^20^ 2200 ng cm^–2^, and PEG:^22^ 800 ng cm^–2^. Also, the performance of Chi-77KS and Chi-77KS/AMOX-coated surfaces in terms of GLO repellency is better than those of other protein-repellent materials reported in the literature, including cellulose:^36^ 1000 ng cm^–2^, carbon film:^61^ 996 ng cm^–2^, ureidopyrimidinone-PEG:^23^ 750 ng cm^–2^, and polysiloxane:^62^ 500 ng cm^–2^.

Compared to the neat PDMS surface, this advantageous performance was maintained with a decrease in protein adsorption, and it is best expressed in the presence of a mixed protein solution, which is very important to study, whereas, in a real situation, not only one protein adsorbs on the surface, but competition between different proteins exists. The significantly higher Voigt mass compared to Δ*f*_3_ observed for functionalized PDMS surfaces is associated with the maximum hydration of the layers upon mixed-protein adsorption. The surface functionalization not only facilitates the rejection of the proteins but also reduces their stability (desorption occurred) during adsorption. The comparison of the samples shows that Chi-77KS/AMOX had the greatest influence on protein adsorption in all cases, probably also due to the presence of the drug AMOX. Discussion regarding the influence of the observed surface parameters on protein adsorption might be concluded as follows: (a) unspecific protein adsorption increased with the hydrophobicity of the surface, (b) increased roughness and water content or hydration of the functionalized layers (as determined by QCM-D H_2_O/D_2_O exchange studies) might lead to lower protein adsorption, and (c) more negative ζ-potential (anionic charge and/or surface hydrophobicity) increased the adsorption affinity, which proves that physical interactions dominate the adsorption.

The results followed the known facts about surface parameters on bacterial adhesion in vitro. It has been shown that bacterial adhesion generally increases with hydrophobicity and decreasing surface energy of abiotic surfaces. However, increased substrate roughness can alter the adhesion of bacteria because it provides a greater area for bacteria to attach to.^63^ Because most bacteria bear a net negative surface charge, adsorption of bacteria is discouraged on negatively charged surfaces, while it is promoted on positively charged surfaces.^64^

### 3.5 “Real” PDMS Samples

The term “real” sample denotes a sample composed of medical-grade material in bulk, rather than the same biomaterial coated on specific holders or carriers (e.g., QCM-D crystals) to test and analyze their properties and so forth. Introduction of “real” samples enabled us to get as close as possible to studying the surface of actual medical materials. Silicone samples in the form of PDMS discs (*A* = 1.2 cm^2^, *h* = 1.5 mm) were chosen for the “real” samples, as they are used commonly in medical devices. While “model” samples showed promising results, “real” samples functionalized with Chi-77KS and Chi-77KS/AMOX in the same way as “model” surfaces were tested, to show the applicability of the developed coatings on “real”, not only “model” surfaces (see Figure 1, right). After surface analyses, we evaluated the antibiofilm properties of the functionalized surfaces.

#### 3.5.1 ATR-FTIR Spectroscopy and Wettability

**Figure 10:**
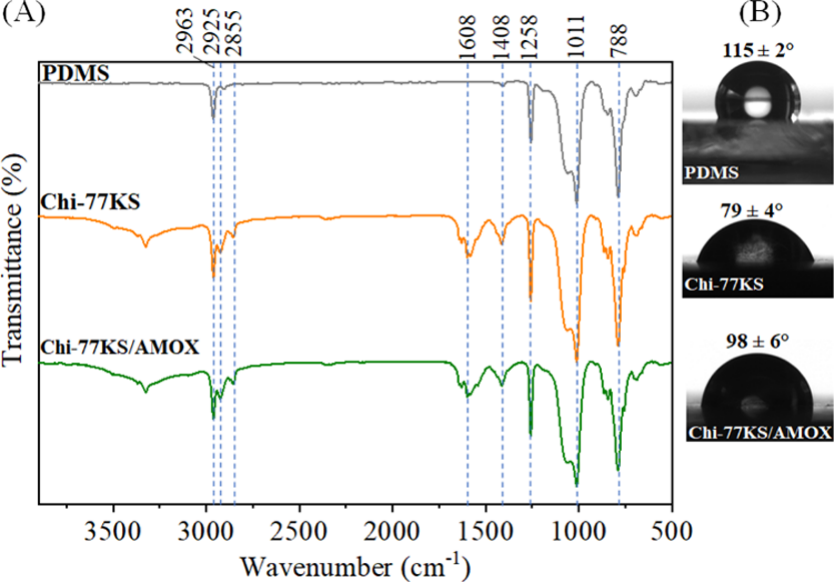
ATR-FTIR spectra (A) and static water contact images (B) of neat PDMS and coated PDMS discs.

ATR-FTIR measurements were performed to confirm the chemical structure and the presence of coatings on the discs’ surfaces. As shown in Figure 10A, PDMS exhibited a peak at 2963 cm^–1^, which corresponds to asymmetric CH_3_ stretching in Si–CH_3_. The peak at 1258 cm^–1^ appeared due to CH_3_ deformation in Si–CH_3_, the peak at 1011 cm^–1^ can be assigned to Si–O–Si stretching, and the peak at 788 cm^–1^ to −CH_3_ rocking and Si–C stretching in Si–CH_3_.^65^ PDMS samples functionalized with Chi-77KS and Chi-77KS/AMOX featured a peak at 3300 cm^–1^, which can be assigned to the N–H stretching vibration in 77KS and hydrogen bonding. The spectra of both Chi-77KS and Chi-77KS/AMOX featured bands that are characteristic of the two, as discussed in ref (24). Both Chi-77KS and Chi-77KS/AMOX spectra exhibited a peak at 1408 cm^–1^ (symmetric −COO^–^ stretching) and peaks at 2855, 2925, and 2955 cm^–1^, which originated from 77KS.^24^ The Chi spectrum featured an intensive band in the 3200–3400 cm^–1^ region, corresponding to a hydrogen bonding and the presence of O–H and N–H groups.^66,67^ Thus, changes in the Chi-77KS and Chi-77KS/AMOX spectra compared to the neat PDMS spectrum confirmed the presence of the adsorbed layer. This can be further verified by the XPS results (see Table 5), which clearly showed the nitrogen element stemming from chitosan in the Chi-77KS coating and also the sulfur and increased content of nitrogen elements for Chi-77KS/AMOX. This is further supported by static water contact angle measurements (Figure 10B).

**Table 5:** Atomic Compositions Obtained from XPS Survey Spectra for “Real” PDMS Discs before and after Functionalization with Chi-77KS and Chi-77KS/AMOX^a^

	atomic percentage (%)				
	C	O	Si	N	S
theoretical	50	25	25		
PDMS	46.80 ± 1.5	28.74 ± 1.2	24.46 ± 1.4		
Chi-77KS	48.69 ± 1.1	24.86 ± 1.1	22.77 ± 1.3	2.24 ± 0.4	
Chi-77KS/AMOX	51.07 ± 1.4	27.26 ± 0.8	17.33 ± 1.4	3.69 ± 0.3	0.65 ± 0.1

a All concentrations are given in atomic %.

Compared to the neat PDMS “model” surfaces with a SCA(H_2_O) of 111 ± 0.6°, the neat PDMS discs as “real” samples exhibited a SCA(H_2_O) of 115 ± 2°. The SCA(H_2_O) of neat PDMS lowered to 79 ± 4° and 98 ± 6° after coating with the Chi-77KS and Chi-77KS/AMOX layers. Even though these values are relatively comparable to the SCA(H_2_O) values obtained for “model” PDMS coated with both Chi-77KS and Chi-77KS/AMOX (see Figure 3), the observed SCA(H_2_O) values are still higher for both functionalized surfaces and PDMS contributes to higher SCA(H_2_O) values to some extent. This is supported by the XPS results (see Table 5), where the presence of silicon is still detected for both functionalized surfaces but in a lower concentration for the Chi-77KS/AMOX coating. This implies that the surfaces of PDMS are less evenly covered by Chi-77KS or Chi-77KS/AMOX. Nevertheless, the observed IR peaks, the presence of N and S from XPS, and improved wettability indicate that the neat PDMS “real” samples are successfully and irreversibly coated with functional layers of Chi-77KS and Chi-77KS/AMOX, which is sufficient to prevent the growth of bacteria or biofilm formation (see Section 3.4.2).

#### 3.5.2 Antibiofilm Capacity of Coated Discs

**Figure 11:**
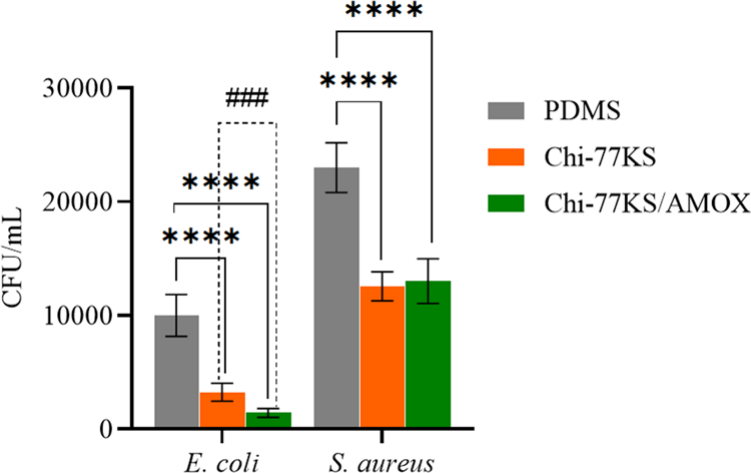
Comparison of difference in bacterial attachment and early biofilm formation on blank and coated PDMS discs after 24 h of incubation for *E. coli* and *S. aureus*. Data analysis was done by one-ANOVA with the Dunnett test, values are presented as ± SD; ****p < 0.05 (compared to negative control PDMS) and by Student’s *t*-test with Mann–Whitney test; ####*p* < 0.05.

Besides the neat PDMS as a control sample, the Chi-77KS and Chi-77KS/AMOX functionalized PDMS discs were used to study bacterial attachment to the surface (biofilm-forming bacteria). The concentration of bacteria (*E. coli* and *S. aureus*) in CFU mL^–1^ that adhered to the PDMS surface was evaluated after 24 h of incubation using the colony-counting method. Because there is no universally accepted standard and clearly defined quality criteria for quantification of bacteria in biofilms,^68^ interpretation of the results remains within the comparison of biofilm depletion between coated (Chi-77KS and Chi-77KS/AMOX) and neat PDMS. The difference in CFU found on the coated sample surface was statistically significant in comparison to the blank samples. All coatings hindered bacterial attachment and, thus, consequently, suppressed biofilm formation. Generally, as noticed from the CFU mL^–1^, *S. aureus* was more inclined to adhere to the surface than *E. coli* (Figure 11).

The amount of *S. aureus* attached to Chi-77KS was found to be 45% lower, and the amount of *E. coli* was 67% lower than on the blank PDMS surface, indicating that the Chi-77KS coating suppressed bacteria attachment and growth successfully. Surprisingly, coating with loaded AMOX (Chi-77KS/AMOX) showed the most significant antibiofilm behavior against *E. coli*, with an 86% lower colony count, while repelling the least bacteria in the case of *S. aureus*, with a 43% decrease in formed colonies (compared to PDMS). Both Chi-77KS and Chi-77KS/AMOX provided beneficial antibiofilm properties for “real” systems, with a significant reduction of bacteria count present on the sample surface when compared to the neat PDMS surface. The antimicrobial nature of the coatings can be attributed to the presence of both cationic Chi and 77KS (anionic and amphiphile) surfactant, which can interact with the cell surface. Their activity against *E. coli* and *S. aureus* strongly depends on how well they can disturb the outer surface of bacteria (microbial cell wall/membrane) whose structural integrity is vital for biological viability. The antimicrobial action depends on the amount of cationic groups (−NH_3_^+^) and their electrostatic interactions with the negatively charged components present on bacterial surface, followed by rupture of the surface to release the intracellular components. In addition, the hydrophobic interactions between the antimicrobial components and components of the membrane wall cannot be avoided. Both these (electrostatic and hydrophobic) interactions result in deformation and subsequent distortion of the cell wall/membrane, which can lead to inhibition of microbial growth and eventually cell death, as reported by other authors.^10^ In the case of Chi-77KS/AMOX coating, the antibiotic AMOX (amphoteric in nature) can act against both Gram-negative (*E. coli*) and Gram-positive (*S. aureus*) microbes by preventing the biosynthesis and repair of the bacterial mucopeptide wall.^69^ Overall, it can be stated that the developed multifunctional coatings containing Chi, 77KS, and AMOX showed the antimicrobial effect not only against Gram-negative *E. coli* comprising bilayers, that is, inner cytoplasmatic and outer membranes but also on Gram-positive *S. aureus* that has a cytoplasmic membrane covered with peptidoglycan.

## 4 Conclusions

In this study, we developed a water-based synergistic PESC consisting of cationic chitosan (Chi), an anionic lysine-based surfactant (77KS), and an amphoteric antibiotic, amoxicillin (AMOX), and applied it to polydimethylsiloxane-based implants in the form of bioactive nanolayers with simultaneous protein-repellent and antimicrobial properties. These bioactive and multifunctional nanocoatings incorporated without (Chi-77KS) and with AMOX (Chi-77KS/AMOX) are created on the “model” and “real” PDMS sample surfaces by effortless QCM-D adsorption and dip-coating techniques. Coating of both formulations under dynamic and ambient conditions resulted in irreversible deposition, smooth morphology, roughness, improved surface coverage, and increased hydrophilicity and water content in comparison to neat PDMS as revealed by QCM-D, atomic force microscopy, and wettability measurements. Compared to negative and positive control samples, i.e., PDMS and PEG-SH, PDMS functionalized with hydrophilic nanolayers of Chi-77KS and Chi-77KS/AMOX showed excellent protein-repellence against all three proteins (bovine serum albumin, fibrinogen, and γ-globulin) and their mixtures. Especially, the Chi-77KS/AMOX nanolayer with the maximum water content (40%), hydration, that is, with a low elastic shear modulus and viscosity exhibited reduced adsorption of proteins in the following order: mixed-protein solution > BSA > FIB > GLO. The bioactive nanolayers exhibiting a negative ζ-potential also displayed an improved reduction of both Gram-positive and Gram-negative bacteria than the neat PDMS. While the reduction of *E. coli* and *S. aureus* is about 80 and 67% with Chi-77KS/AMOX, the Chi-77KS without AMOX showed approximately 40% reduction of both microbes. Given the multifunctionalities and easy application technique, the bioactive coatings reported in this study can be applied to not only silicone-based medical implants but also to other standard devices used in clinical or biomedical applications (e.g., surgical reconstructive components, heart pumps, and so forth).

## Supporting Information Available

Potentiometric charge titration curves, charges, and isoelectric points of proteins (BSA, FIB, and GLO) and QCM-D Δ*f*_3_ of the adsorption of PEG-SH, protein adsorption on the latter surface, and on the neat PDMS and Chi-77KS- or Chi-77KS/AMOX-coated surfaces (PDF)
