# Supplementary material for: Bioactive Functional Nanolayers of Chitosan–Lysine Surfactant with Single- and Mixed-Protein-Repellent and Antibiofilm Properties for Medical Implants
Source: ACS Appl Mater Interfaces. 2021 May 17;13(20):23352–68. doi: 10.1021/acsami.1c01993 (PMC8289181; doi:10.1021/acsami.1c01993)
Supplement: Supplementary file 1 — am1c01993_si_001.pdf [file am1c01993_si_001.pdf]

# Supporting Information

# Bioactive Functional Nanolayers of Chitosan-Lysine Surfactant with Single- and Mixed-Protein-Repellent and Antibiofilm Properties for Medical Implants

*Urban Ajdnik*[†], *Lidija Fras Zemljič*[†], *Olivija Plohl*[†], *Lourdes Pérez*[‡], *Janja Trček*[β], *Matej Bračič*[†], *Tamilselvan Mohan*[$*]

[†]Faculty of Mechanical Engineering, Institute of Engineering Materials and Design, Laboratory for Characterization and Processing of Polymers, University of Maribor, 2000 Maribor, Slovenia

[‡]Institute for Advanced Chemistry of Catalonia IQAC-CSIC, Department of Surfactants and Nanobiotechnology, 08034 Barcelona, Spain

[β]Faculty of Natural Sciences and Mathematics, Department of Biology, University of Maribor, 2000 Maribor, Slovenia

[$]Institute for Chemistry and Technology of Biobased Systems (IBioSys), Graz University of Technology, 8010 Graz, Austria

**KEYWORDS:** silicone implants, protein-repellent, antimicrobial, chitosan, lysine, bioactive coatings, adsorption, QCM-D

## Potentiometric titration

Briefly, pH-potentiometric titration of amoxicillin, bovine serum albumin (BSA), fibrinogen (FIB) and γ-globulin (GLO) were determined as follows. The titration cell was filled with analyte solution of a known concentration and titrated (T70, Mettler Toledo, Greifensee, Switzerland) in the pH region 2.5–11.0 using 0.1 M HCl and 0.1 M KOH. Measurements were performed in an inert $N_2$ atmosphere and at an ionic strength of 0.1 M KCl using InLab Routine pH electrode (Mettler Toledo, Giessen, Germany). The detailed description of the method can be found elsewhere [1, 2].

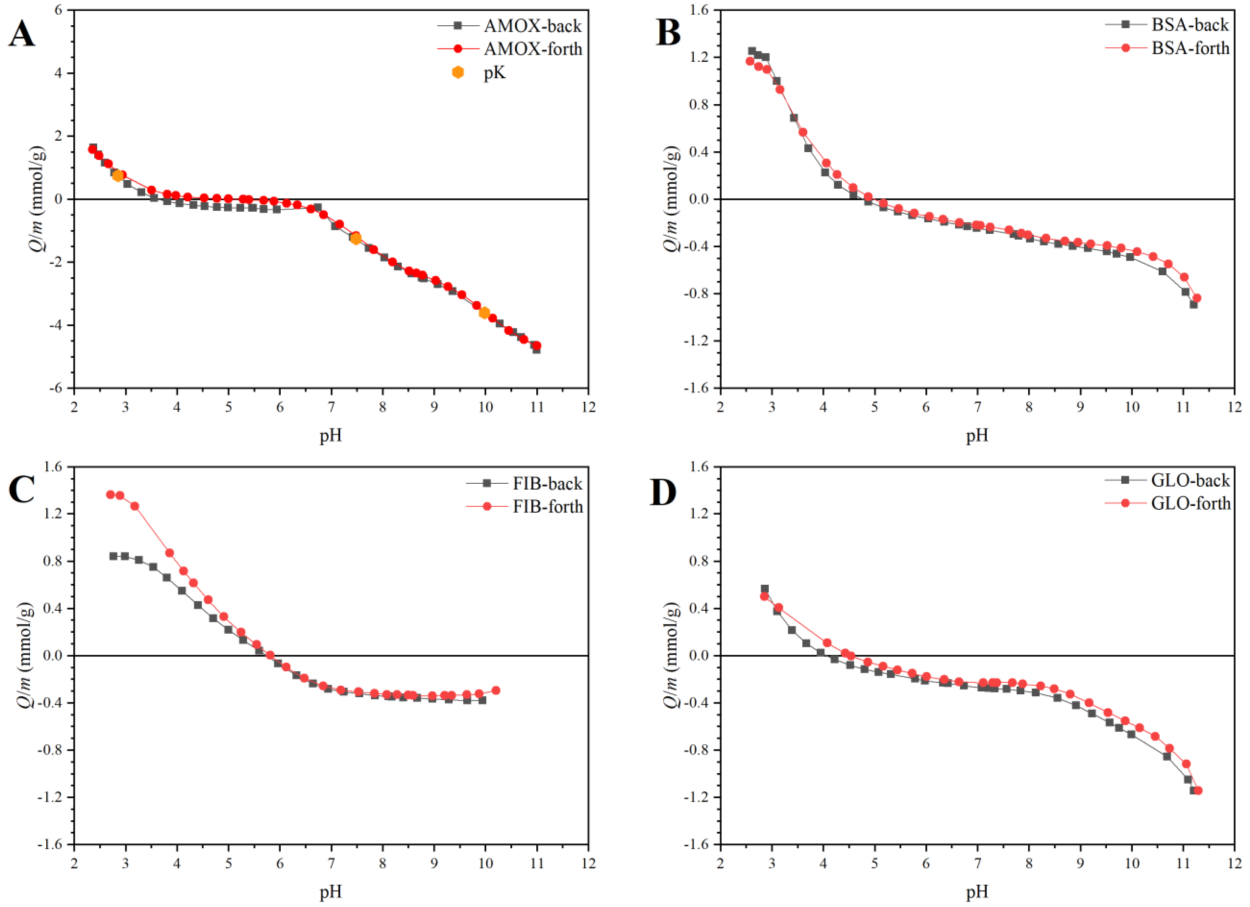


**Figure S1.** Potentiometric titration curves of (A) AMOX, (B) BSA, (C) FIB and (D) GLO.

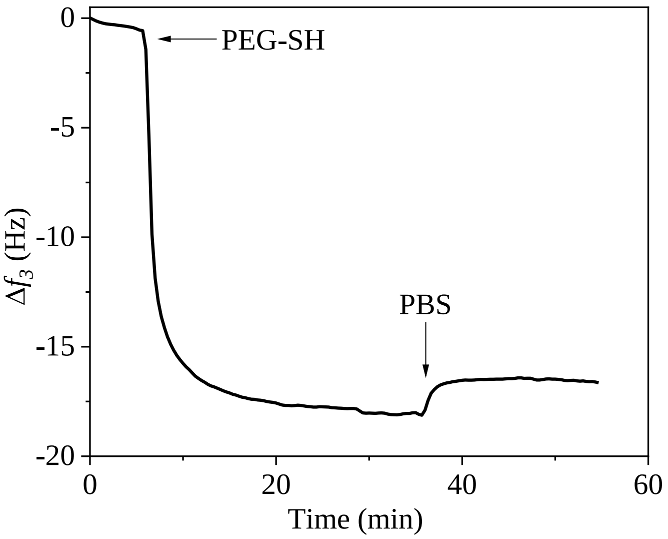


**Figure S2**. QCM-D adsorption of poly(ethylene glycol) methyl ether thiol (PEG-SH) onto the neat gold surface.

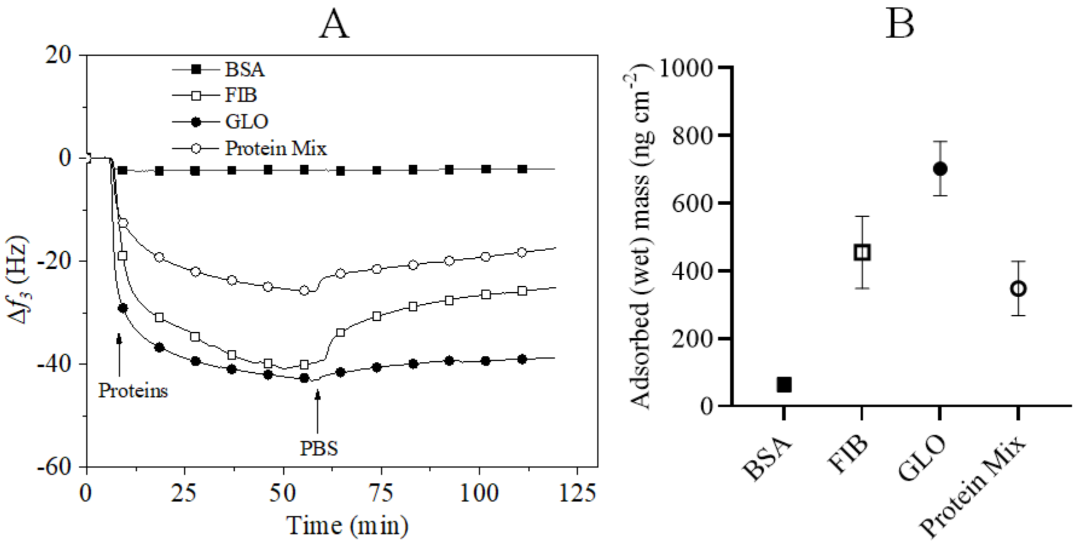


**Figure S3**. (A) QCM-D adsorption (A) and (B) comparison of adsorbed (wet) masses of proteins on PEG-SH (negative control) coated surfaces.

**Table S1.** Protein charges (pH = 7.4) and pI of BSA, FIB and GLO.

| **protein** | **$Q/m$ [mmol g$^{-1}$]** | **pI** |
|---|---|---|
| BSA | –0.26 | 4.9 |
| FIB | –0.31 | 5.8 |
| GLO | –0.25 | 4.3 |

**Table S2.** BSA frequency change ($\Delta f_3$), dissipation change ($\Delta D_3$), desorption ratio $\Delta f_B/\Delta f_A$ and $\Delta D_3/\Delta f_3$ ratio (A is $\Delta f_3$ and $\Delta D_3$ before rinsing, B is the final $\Delta f_3$ and $\Delta D_3$ after rinsing), and viscoelastic properties of the adsorbed BSA layer.

| **BSA** | **$\Delta f_3$** [Hz] | | **$\Delta f_B/\Delta f_A$** [%] | **$\Delta D_3$** [$10^{-6}$] | | **$\Delta D_3/\Delta f_3$** [$10^{-7}$ Hz$^{-1}$] | |
|---|---|---|---|---|---|---|---|
| | **A** | **B** | | **A** | **B** | **A** | **B** |
| **PDMS** | –35.5 ± 2.0 | –34.3 ± 1.7 | 96.6 | 2.2 ± 0.3 | 1.8 ± 0.4 | 0.6 | 0.5 |
| **Chi-77KS** | –31.3 ± 1.7 | –30.8 ± 3.0 | 98.4 | 1.7 ± 0.1 | 2.4 ± 0.8 | 0.5 | 0.8 |
| **Chi-77KS/AMOX** | –10.8 ± 1.0 | –13.5 ± 1.5 | 125.0 | 0.4 ± 0.1 | 1.7 ± 0.1 | 0.4 | 1.3 |
| | Viscoelastic properties | | | | | | |
| | **$\Gamma_{QCM}$ wet** [ng m$^{-2}$] | | **$h_f$** [nm] | **$\eta_f \times 10^{-3}$** [Ns m$^{-2}$] | | **$\mu_f \times 10^4$** [N m$^{-2}$] | |
| **PDMS** | 639.09 ± 8.02 | | 6.39 ± 1.04 | 0.004 ± 0.001 | | 97.57 ± 5.21 | |
| **Chi-77KS** | 650.10 ± 5.23 | | 6.50 ± 0.98 | 0.002 ± 0.000 | | 25.56 ± 2.96 | |
| **Chi-77KS/AMOX** | 487.83 ± 2.48 | | 4.88 ± 0.35 | 0.002 ± 0.000 | | 6.44 ± 1.25 | |

**Table S3.** FIB frequency change ($\Delta f_3$), dissipation change ($\Delta D_3$), desorption ratio $\Delta f_B/\Delta f_A$ and $\Delta D_3/\Delta f_3$ ratio (A is $\Delta f_3$ and $\Delta D_3$ before rinsing, B is the final $\Delta f_3$ and $\Delta D_3$ after rinsing), and viscoelastic properties of the adsorbed FIB layer.

| **FIB** | **$\Delta f_3$** [Hz] | | **$\Delta f_B/\Delta f_A$** [%] | **$\Delta D_3$** [$10^{-6}$] | | **$\Delta D_3/\Delta f_3$** [$10^{-7}$ Hz$^{-1}$] | |
|---|---|---|---|---|---|---|---|
| | **A** | **B** | | **A** | **B** | **A** | **B** |
| **PDMS** | –92.1 ± 3.0 | –78.3 ± 2.7 | 85.0 | 10.0 ± 1.3 | 8.5 ± 1.4 | 1.1 | 1.1 |
| **Chi-77KS** | –25.1 ± 2.0 | –20.2 ± 2.7 | 80.5 | 1.0 ± 0.2 | –0.1 ± 0.1 | 0.4 | 0.1 |
| **Chi-77KS/AMOX** | –18.2 ± 2.0 | –14.3 ± 1.1 | 78.6 | 1.4 ± 0.3 | 1.9 ± 0.2 | 0.8 | 1.3 |
| | Viscoelastic properties | | | | | | |
| | **$\Gamma_{QCM}$ wet** [ng m$^{-2}$] | | **$h_f$** [nm] | **$\eta_f \times 10^{-3}$** [Ns m$^{-2}$] | | **$\mu_f \times 10^4$** [N m$^{-2}$] | |
| **PDMS** | 1467.70 ± 18.62 | | 14.68 ± 2.37 | 0.002 ± 0.000 | | 59.71 ± 8.91 | |
| **Chi-77KS** | 355.28 ± 7.37 | | 3.55 ± 0.25 | 0.002 ± 0.000 | | 1738.00 ± 95.24 | |
| **Chi-77KS/AMOX** | 349.31 ± 2.86 | | 3.49 ± 0.08 | 0.002 ± 0.000 | | 19.74 ± 2.68 | |

**Table S4.** GLO frequency change ($\Delta f_3$), dissipation change ($\Delta D_3$), desorption ratio $\Delta f_B/\Delta f_A$ and $\Delta D_3/\Delta f_3$ ratio (A is $\Delta f_3$ and $\Delta D_3$ before rinsing, B is the final $\Delta f_3$ and $\Delta D_3$ after rinsing), and viscoelastic properties of the adsorbed GLO layer.

| **GLO** | **$\Delta f_3$** [Hz] | | **$\Delta f_B/\Delta f_A$** [%] | **$\Delta D_3$** [$10^{-6}$] | | **$\Delta D_3/\Delta f_3$** [$10^{-7}$ Hz$^{-1}$] | |
|---|---|---|---|---|---|---|---|
| | **A** | **B** | | **A** | **B** | **A** | **B** |
| **PDMS** | –46.9 ± 2.0 | –48.8 ± 1.7 | 104.1 | 2.6 ± 0.7 | 3.5 ± 0.9 | 0.6 | 0.7 |
| **Chi-77KS** | –38.6 ± 1.9 | –39.8 ± 1.6 | 103.1 | –2.3 ± 0.9 | 1.0 ± 0.1 | 0.6 | 0.3 |
| **Chi-77KS/AMOX** | –33.8 ± 3.0 | –35.3 ± 4.0 | 104.4 | 0.5 ± 0.1 | 4.5 ± 1.1 | 0.1 | 1.3 |
| Viscoelastic properties | | | | | | | |
| | **$\Gamma_{QCM}$ wet** [ng m$^{-2}$] | | **$h_f$** [nm] | **$\eta_f \times 10^{-3}$** [Ns m$^{-2}$] | | **$\mu_f \times 10^4$** [N m$^{-2}$] | |
| **PDMS** | 978.23 ± 12.55 | | 9.78 ± 1.56 | 0.002 ± 0.000 | | 122.98 ± 6.77 | |
| **Chi-77KS** | 776.24 ± 20.87 | | 7.76 ± 2.01 | 0.004 ± 0.001 | | 52.59 ± 2.87 | |
| **Chi-77KS/AMOX** | 629.14 ± 8.17 | | 6.29 ± 1.83 | 0.001 ± 0.000 | | 134.14 ± 8.16 | |

**Table S5.** Protein mixture frequency change ($\Delta f_3$), dissipation change ($\Delta D_3$), desorption ratio $\Delta f_B/\Delta f_A$ and $\Delta D_3/\Delta f_3$ ratio (A is $\Delta f_3$ and $\Delta D_3$ before rinsing, B is the final $\Delta f_3$ and $\Delta D_3$ after rinsing), and viscoelastic properties of the adsorbed BSA layer.

| **Protein mixture** | **$\Delta f_3$** [Hz] | | **$\Delta f_B/\Delta f_A$** [%] | **$\Delta D_3$** [$10^{-6}$] | | **$\Delta D_3/\Delta f_3$** [$10^{-7}$ Hz$^{-1}$] | |
|---|---|---|---|---|---|---|---|
| | **A** | **B** | | **A** | **B** | **A** | **B** |
| **PDMS** | –126.2 ± 4.0 | –104.7 ± 6.2 | 83.0 | 26.7 ± 2.1 | 20.1 ± 1.3 | 2.1 | 1.9 |
| **Chi-77KS** | –45.1 ± 3.4 | –56.4 ± 3.3 | 125.1 | 8.3 ± 2.0 | 18.1 ± 1.1 | 1.8 | 3.2 |
| **Chi-77KS/AMOX** | –22.7 ± 2.2 | –18.1 ± 1.4 | 79.7 | 4.8 ± 1.2 | 7.1 ± 0.9 | 2.1 | 3.9 |
| | Viscoelastic properties | | | | | | |
| | **$\Gamma_{QCM}$ wet** [ng m$^{-2}$] | | **$h_f$** [nm] | **$\eta_f \times 10^{-3}$** [Ns m$^{-2}$] | | **$\mu_f \times 10^4$** [N m$^{-2}$] | |
| **PDMS** | 2662.60 ± 25.89 | | 26.63 ± 2.86 | 0.002 ± 0.000 | | 13.69 ± 1.52 | |
| **Chi-77KS** | 2550.40 ± 20.67 | | 25.50 ± 1.62 | 0.002 ± 0.000 | | 7.26 ± 0.86 | |
| **Chi-77KS/AMOX** | 2307.60 ± 18.66 | | 23.08 ± 2.08 | 0.001 ± 0.000 | | 1.76 ± 0.05 | |

## REFERENCES


[1] D. Čakara, L. Fras, M. Bračič, and K. S. Kleinschek, "Protonation behavior of cotton fabric with irreversibly adsorbed chitosan: A potentiometric titration study," *Carbohydrate Polymers,* vol. 78, no. 1, pp. 36-40, 2009/08/04/ 2009.

[2] L. F. Zemljič, D. Čakara, N. Michaelis, T. Heinze, and K. Stana Kleinschek, "Protonation behavior of 6-deoxy-6-(2-aminoethyl)amino cellulose: a potentiometric titration study," *Cellulose,* vol. 18, no. 1, pp. 33-43, 2011/02/01 2011.
